# Cytotoxic evaluation and chemical investigation of tomatoes from plants (*Solanum lycopersicum* L.) grown in uncontaminated and experimentally contaminated soils

**DOI:** 10.1038/s41598-022-13876-w

**Published:** 2022-07-29

**Authors:** Chiara Russo, Daniela Barone, Margherita Lavorgna, Concetta Piscitelli, Marcella Macaluso, Severina Pacifico, Simona Piccolella, Antonio Giordano, Marina Isidori

**Affiliations:** 1 Department of Environmental, Biological and Pharmaceutical Sciences and Technologies, University of Campania “Luigi Vanvitelli”, Via Vivaldi 43, 81100 Caserta, Italy; 2 Cell Biology and Biotherapy Unit, Istituto Nazionale Tumori ‑ IRCCS ‑ Fondazione G. Pascale, 80131 Napoli, Italy; 3 Department of Biology, College of Science and Technology, Sbarro Institute for Cancer Research and Molecular Medicine, Temple University, Philadelphia, PA USA

**Keywords:** Cancer, Cell biology, Chemical biology, Plant sciences, Stem cells

## Abstract

The aim of this study was to evaluate the cytotoxic activity and the chemical composition of the tomato extracts coming from, Pomodoro Giallo and San Marzano Cirio 3, and then to evaluate the potential changes when plants were grown in soils contaminated by cadmium, chromium and lead. Extracts were investigated by UHPLC-HRMS and UV–Vis. Cell viability (CellTiter-Glo Luminescent assay), enzyme aldehyde dehydrogenase activity (ALDEFLOUR Assay), cell cycle progression (Accuri C6 Flow Cytometer), apoptosis and necrosis (Annexin V-FITC assay) were evaluated on two gastric cancer (AGS and NCI-N87) and two colorectal cancer (HT-29 and HCT 116) cell lines. Different content of polyphenol and carotenoid constituents was observed. Extracts from uncontaminated soil induced cytotoxic activity towards all selected cancer cells, while extracts coming from contaminated soils showed the aberrant phenotype increased in colorectal cancer cells. Chloroform extracts exerted the highest cytotoxic activity. AGS and HT-29 were the most sensitive to cell cycle arrest and to apoptosis. No necrotic effect was observed in HCT 116. The contrasting effects on cancer cells were observed based on tomato variety, the extract polarity, heavy metal identity, and tested cell line. The investigation of potential adverse health effects due to Cd in the fruits should be explored.

## Introduction

Among fruits and vegetables, tomatoes play an important role in the prevention of various degenerative diseases^1,2^ exerting beneficial effects on human health for their richness in phytochemicals, known for their antioxidant^3^ anti-inflammatory^4,5^, anti-genotoxic, anti-mutagenic^6,7^, anti-proliferative activities^1,8^.

In Campania region (Italy), tomato plants are mainly cultivated in Agro Nocerino Sarnese, area of environmental concern because of the pollution of the Sarno River that crosses through it. The Sarno is considered one of the most disturbed aquatic ecosystems in Europe due to the uncontrolled industrial and urban spillage^9^ that mostly causes a strong pollution of freshwater that very often are used for irrigation of fields. Montuori and co-authors^10^ demonstrated that the Sarno river contributes to contaminate the Tyrrhenian Sea with metals and polycyclic aromatic hydrocarbons. In Parrella et al.^11^, the porewaters of the Sarno and Volturno rivers showed mutagenicity/genotoxicity. Cicchella et al.^12,13^ demonstrated that soils and sediments from this area were polluted by Cr, Hg, As, Be, Sn and Cu which can be easily adsorbed in the soil and in the river bed because of the geological nature of that area. It is well-known that heavy metals are persistent and can be absorbed by plants, reaching fruits and, through food chain, they can enter the human body, predisposing to diseases and promoting cell transformation^14,15^, with noticeable increase in lung, stomach, bladder and brain cancers.

The aim of the present study has been to evaluate the cytotoxic activity of tomato fruit extracts towards the selected Cancer Stem like Cells (CSCs) and then to appraise if this activity underwent modifications when tomatoes were from plants grown in soils contaminated by heavy metals. CSCs represent a subpopulation of cells able to self-renew and differentiate to repopulate the tumor mass. Since chemotherapeutics may not completely eliminate CSCs, there is an increasing interest in isolating and identifying natural compounds able to target CSCs directly or indirectly, by affecting protein expression of drug resistance associated genes or/and signaling pathways. Studies suggest that natural compounds have potential beneficial effects on cancer treatment as they are able to reduce cell proliferation, to induce cell cycle arrest and apoptosis, and to target CSCs self-renewal through specific molecular mechanisms^16–19^.

In this study, two tomato varieties have been used, Pomodoro Giallo (PG) and San Marzano Cirio 3 (PR), already known to be very rich in beneficial compounds compared to other tomato varieties (e.g. San Marzano, Corbarino di Accadia, Nero di Sicilia) investigated in our previous studies^1,2^. The extracts obtained were preliminarily investigated for their chemical composition by means of ultra-high-pressure liquid chromatography (UHPLC) coupled to electrospray ionization quadrupole time-of-flight tandem mass spectrometry (ESI-QqTOF MS/MS) in negative ion mode and UV–Vis spectroscopy. In particular, the extracts of PG and PR came from plants grown in uncontaminated and experimental contaminated soils by chromium (Cr), lead (Pb) or cadmium (Cd) in order to generate perceptible changes in plant metabolism^20^. The cytotoxic activity has been evaluated on four human CSCs such as gastric cancer (AGS and NCI-N87) cells and colorectal cancer (HT-29 and HCT 116) cells^21,22^, chosen because stomach cancer (769,000 deaths in 2020) and colorectal cancer (935,000 deaths in 2020) are among the main causes of tumor death worldwide [https://www.who.int/news-room/fact-sheets/detail/cancer].

Different cytotoxic analysis approaches were used: the CellTiter-Glo Luminescent assay for evaluating the antiproliferative activity, the ALDEFLOUR Assay for evaluate ALDH-positive cell population, the Accuri C6 Flow Cytometer to study the cell cycle and, finally, the Annexin V-FITC assay to evaluate the induction of apoptosis and necrosis in tumor cells.

## Results

### Tomato fruits extracts chemical profiling

In order to highlight the impact of heavy metal contamination on specialized metabolites in tomato fruits, these latter underwent biphasic extraction^23^ to preliminarily obtain a hydroalcoholic and a lipophilic extract, whose chemical characterization was mainly by means of UHPLC-ESI-QqToF/MS and MS/MS techniques (Tables 1, 2, 3). Based on previous evidence about the presence of polyphenol compounds, the negative ion acquisition mode was of choice. Moreover, UV–Vis spectroscopy was used to detect the presence of carotenoids in the lipophilic samples.

**Table 1:** UHPLC-ESI-QqToF/MS and MS/MS data useful for tentative identification of non-phenolic compounds in hydro-alcoholic extracts (base peaks in MS/MS spectra are reported in bold; RT = retention time; RDB = ring and double bond value). MS/MS spectra are reported as Supplementary Information.

Peak n	RT (min)	Tentative assignment	Formula	[M-H]^-^ found (*m/z*)	Error (ppm)	RDB	MS/MS fragment ions (*m/z*)
1	0.322	Aspartic acid	C_4_H_7_NO_4_	132.0307	3.5	2	132.0311; 115.0046; **88.0417**
2	0.344	Glutamic acid	C_5_H_9_NO_4_	146.0463	2.9	2	146.0462; 128.0353; **102.0565**
3	0.399	Citric acid	C_6_H_8_O_7_	191.0201	2.0	3	191.0193; 129.0193; 111.0091; **87.0092**; 85.0298
4	0.937	Methylcitric acid	C_7_H_10_O_7_	205.0358	2.1	3	143.0352; **111.0092**; 87.0090
5	1.140	4-Hydroxyphenyl β-sophoroside	C_18_H_26_O_12_	433.1374	2.4	6	433.1374; 271.0829; 221.0669; 161.0460; 109.0297; **108.0220**
6	1.188	Phenylalanine	C_9_H_11_NO_2_	164.0725	4.9	5	**164.0718**; 147.0451; 103.0555; 89.0249
7	1.578	Hexosyl phenylalanine	C_15_H_21_NO_7_	326.1246	0.2	6	236.0926; **164.0722**; 147.0455; 103.0559
9	2.944	Tryptophan	C_11_H_12_N_2_O_2_	203.0832	2.9	7	203.0813; 142.0673; **116.0505**
10	2.951	Leucinopine	C_11_H_19_NO_6_	260.1144	1.7	3	**260.1148**; 242.1042; 214.1088; 198.1140; 196.0983; 170.1183; 144.0671; 132.0672; 114.0563; 100.0770; 83.0505
11	3.430	Pantothenic acid 4’-*O*-glucoside	C_15_H_27_NO_10_	380.1571	2.8	3	**380.1583**; 362.1462; 308.1358; 161.0452; 146.0830
14	4.073	Unknown	C_18_H_28_O_10_	403.1621	2.6	5	**403.1635**; 223.0977; 179.1085; 161.0979; 149.0977; 119.0357; 113.0245; 101.0247; 89.0247
20	4.752	*N*-phenylacetyl aspartic acid	C_12_H_13_NO_5_	250.0725	1.6	7	250.0714; 132.0310; 115.0042; **88.0410**
22	4.832	Unknown	C_18_H_28_O_10_	403.1620	2.6	5	**403.1636**; 385.1529; 241.1087; 221.0676; 179.1080; 163.0769; 161.0978; 161.0459; 145.0662; 113.0249; 101.0248
26	5.507	Tuberonic acid hexoside 1	C_18_H_28_O_9_	387.1673	2.2	5	387.1686; **163.1137**; 119.0362; 113.0254; 101.0255; 89.0254
27	5.777	Tuberonic acid hexoside 2	C_18_H_28_O_9_	387.1673	2.2	5	**387.1665**; 163.1126; 119.0345; 113.0240; 101.0243; 89.0242
29	6.106	Tuberonic acid hexoside 3	C_18_H_28_O_9_	387.1668	1.9	5	**387.1686**; 369.1570; 207.1037; 163.1135; 119.0359; 113.0251; 101.0249; 89.0255
32	7.079	8-Hydroxy-2,7-dimethylocta-4,6-dienoic acid hexoside	C_18_H_28_O_10_	403.1616	1.6	5	403.1619; 283.1189; **241.1079**; 197.1183; 181.0863

**Table 2:** UHPLC-ESI-QqToF/MS and MS/MS data useful for tentative identification of phenolic compounds in hydro-alcoholic extracts (base peaks in MS/MS spectra are reported in bold; RT = retention time; RDB = ring and double bond value). MS/MS spectra are reported as Supplementary Information.

Peak no	RT (min)	Tentative assignment	Formula	[M-H]^−^ found (*m/z*)	Error (ppm)	RDB	MS/MS fragment ions (*m/z*)
**8**	1.830	Hydroxybenzoic acid hexoside	C_13_H_16_O_8_	299.0769	− 1.1	6	**137.0249**; 93.0349
**12**	3.554	3-CQA	C_16_H_18_O_9_	353.0887	2.5	8	353.0894; **191.0567**; 179.0354; 135.0452; 134.0369
**13**	4.009	3-CQA dihexoside	C_28_H_28_O_19_	677.1956	3.2	10	677.1974; 515.1431; 353.0883; 323.0772; **191.0561**; 179.0345; 161.0246
**15**	4.266	*p*-coumaric acid hexoside 1	C_15_H_18_O_8_	325.0929	0.0	7	163.0409; **119.0510**
**16**	4.363	Caffeic acid hexoside 1	C_15_H_18_O_9_	341.0884	1.7	7	281.0669; 251.0556; 221.0454; 179.0351; 161.0241; **135.0454**; 134.0375
**17**	4.459	Dihydrocaffeic acid hexoside 1	C_15_H_20_O_9_	343.1034	− 0.2	6	343.1011; **181.0504**; 137.0608; 135.0451; 121.0295; 119.0507; 109.0295
**18**	4.535	3-CQA hexoside 1	C_22_H_28_O_14_	515.1415	1.7	9	515.1441; 353.0882; **191.0565**; 179.0347
**19**	4.598	Dihydrocaffeic acid hexoside 2	C_15_H_20_O_9_	343.1038	1.0	6	343.1049; **181.0516**; 137.0617; 135.0454; 121.0299; 119.0508; 109.0300
**21**	4.753	*p*-coumaric acid hexoside 2	C_15_H_18_O8	325.0932	0.9	7	163.0402; **119.0506**
**23**	4.888	Caffeic acid hexoside 2	C_15_H_18_O_9_	341.0886	2.3	7	341.0889; 281.0672; 251.0562; 221.0456; 179.0354; 161.0243; **135.0456**; 134.0376
**24**	5.150	3-CQA hexoside 2	C_22_H_28_O_14_	515.1428	4.2	9	515.1433; 323.0774; **191.0557**; 179.0349; 161.0241
**25**	5.180	4-CQA	C_16_H_18_O_9_	353.0888	2.8	8	353.0902; 191.0570; 179.0360; **173.0464**; 135.0456; 134.0379
**28**	5.883	5-CQA	C_16_H_18_O_9_	353.0885	2.0	8	**191.0567**
**30**	6.430	Quercetin dihexosyl deoxyhexoside	C_33_H_40_O_21_	771.2015	3.3	14	**771.2039**; 609.1501; 463.0904; 462.0825; 343.0459; 301.0359; 300.0281; 299.0200; 271.0249
**31**	6.620	Kaempferol dihexosyl pentosyl deoxyhexoside	C_38_H_48_O_24_	887.2473	1.3	15	887.2541; **725.1978**; 285.0403; 284.0328
**33**	7.883	Quercetin pentosyl rutinoside	C_32_H_38_O_20_	741.1917	4.5	14	741.1947; 609.1499; 301.0362; **300.0285**; 271.0254; 255.0302
**34**	8.094	Rutin	C_27_H_30_O_16_	609.1492	5.1	13	609.1514; 301.0365; **300.0285**; 271.0254; 255.0304; 243.0302; 178.9988; 151.0037
**35**	8.328	Kaempferol pentosyl rutinoside	C_32_H_38_O1_9_	725.1959	3.4	14	**725.1999**; 593.1558; 575.1444; 327.0519; 285.0404; 284.0331; 255.0296; 227.0347
**36**	8.466	3,4-diCQA	C_25_H_24_O_12_	515.1208	2.5	14	515.1237; 353.0893; 335.0788; 191.0567; 179.0354; **173.0460**; 161.0249; 155.0354; 135.0457
**37**	8.661	3,5-diCQA	C_25_H_24_O_12_	515.1208	2.5	14	353.0900; **191.0568**; 179.0356; 135.0456
**38**	8.673	12-*O*-(caffeoylhexosyl) jasmonate 1	C_27_H_34_O_12_	549.2002	4.5	11	549.2000; 489.1775; **387.1664**; 179.0345; 163.1124; 161.0242; 133.0295
**39**	8.675	Kaempferol rutinoside	C_27_H_30_O_15_	593.1540	4.7	13	593.1551; 327.0503; **285.0403**; 284.0323; 255.0292; 227.0340
**40**	9.093	1,4-diCQA	C_25_H_24_O_12_	515.1212	3.3	14	515.1226; 353.0888; 191.0566; 179.0355; **173.0459**; 135.0449
**41**	9.101	12-*O*-(caffeoylhexosyl) jasmonate 2	C_27_H_34_O_12_	549.2002	4.5	11	549.2021; 489.1785; **387.1683**; 179.0353; 163.1133; 161.0251; 133.0302
**42**	9.413	Caffeic acid derivative	C_23_H_30_O_12_	497.1687	4.5	9	497.1700; 257.1026; **179.0351**; 135.0449
**43**	9.478	Quercetin *p*-coumaroyl deoxyhexosyl hexosyl pentoside	C_41_H_44_O_22_	887.2283	3.6	20	**887.2322**; 741.1937; 723.1832; 301.0350; 300.0274; 271.0236
**44**	9.827	4,5-diCQA	C_25_H_24_O_12_	515.1212	3.3	14	515.1226; 353.0888; 191.0566; 179.0358; **173.0462**; 135.0446
**45**	9.838	Caffeic acid derivative	C_20_H_26_O_10_	425.1467	3.2	8	425.1470; **179.0352**; 135.0454
**46**	10.011	Dihydrocaffeic acid derivative	C_20_H_28_O_10_	427.1621	2.6	7	427.1634; **181.0514**; 137.0614; 135.0457; 121.0300; 119.0499; 109.0299
**47**	10.183	*p*-Coumaric acid derivative	C_20_H_26_O_9_	409.1511	1.7	8	409.1535; 205.0518; 163.0404; **119.0504**
**48**	10.658	*p*-Coumaric acid derivative	C_20_H_26_O_9_	409.1513	2.2	8	163.0398; **119.0501**
**49**	10.836	Tri-CQA	C_34_H_30_O_15_	677.1526	2.1	20	677.1571; **515.1230**; 497.1122; 353.0893; 335.0777; 255.0680; 191.0567; 179.0358; 173.0463; 161.0248; 135.0456

**Table 3:** UHPLC-ESI-QqToF/MS and MS/MS data useful for metabolite tentative identification in lipophilic extracts (base peaks in MS/MS spectra are reported in bold; RT = retention time; RDB = ring and double bond value). MS/MS spectra are reported as Supplementary Information.

Peak no	RT (min)	Tentative assignment	Formula	[M-H]^−^ found (*m/z*)	Error (ppm)	RDB	MS/MS fragment ions (*m/z*)
1’	1.878	*N*-feruloyloctopamine	C_18_H_19_NO_5_	328.1188	− 0.8	10	328.1199; 310.1092; 295.0854; 252.0779; **161.0247**; 133.0531
2’	3.520	*N*-feruloyltyramine	C_18_H_19_NO_4_	312.1240	− 0.4	10	**312.1234**; 297.0991; 253.0836; 190.0498; 178.0509; 148.0523; 135.0450
3’	4.818	Trihydroxy-octadecenoic acid	C_18_H_34_O_5_	329.2329	− 1.4	2	**329.2333**; 311.2225; 293.2107; 229.1446; 211.1343; 171.1031; 139.1132
4’	5.177	Trihydroxy-octadecadienoic acid	C_18_H_32_O_5_	327.2184	2.1	3	327.2185; 309.2070; 291.1974; 209.1182; **171.1029**; 155.1080; 137.0973
5’	6.643	Linolenoyl-glycerol-3-phosphate	C_21_H_37_O_7_P	431.2196	− 1.9	3	431.2198; 277.2147; **152.9951**; 96.9687
6’	6.655	Hydroxy-linoleic acid	C_18_H_32_O_3_	295.2273	− 1.9	3	295.2276; **277.2169**; 195.1385; 183.1026; 171.1019
7’	6.875	Linoleoyl-glycerol 3-phosphate	C_21_H_39_O_7_P	433.2354	− 1.5	3	433.2350; 279.2321; **152.9959**; 96.9701
8’	7.152	Palmitoyl-glycerol 3-phosphate	C_19_H_39_O_7_P	409.2348	− 3.1	1	409.2351; 255.2316; **152.9964**; 96.9705
9’	7.156	Linoleoyl-lysophosphatidic acid monomethyl ester	C_22_H_41_O_7_P	447.2501	− 3.6	3	447.2517; **279.2321**; 167.0111
10’	7.346	Hydroxy-palmitic acid	C_16_H_32_O_3_	271.2276	− 1.0	1	271.2278; **225.2221**
11’	7.347	Linolenic acid	C_18_H_30_O_2_	277.2169	− 1.5	4	**277.2171**
12’	7.599	Linoleic acid	C_18_H_32_O_2_	279.2325	− 1.6	3	**279.2329**
13’	7.781	Palmitic acid	C_16_H_32_O_2_	255.2327	− 1.0	1	**255.2333**

### Mass spectrometric characterization of hydroalcoholic extract constituents

Forty-nine compounds, among which diverse amino acids and organic acids, were part of the hydroalcoholic fraction of studied tomato fruits. Beyond aspartic acid (1), glutamic acid (2), phenylalanine (6), its hexosyl derivative (7), and tryptophan (9), the *N*-(1-carboxy-3-methylbutyl) derivative of glutamic acid (10), also known as leucinopine, and a *N*-phenylacetyl aspartic acid (20) were tentatively identified. Citric acid (3), previously reported as the main organic acid in tomato^24^, and its methyl derivative (4) were also present. Furthermore, it was tentatively identified a dihexosyl hydroxyphenol (5), likely 4-hydroxyphenyl β-d-glucopyranosyl-(1 → 2)-β-d-glucopyranoside, which was while ago isolated from *L. esculentum*^25^.

The great part of hydroalcoholic extract was constituted by hydroxycinnamic acid depsides belonging to chlorogenic acid family, and differing for the number of caffeoyl moieties and the esterification site on the quinate moiety. In particular, TOF–MS/MS data allowed compounds 12, 25 and 28 to be distinguished as 3-, 4- and 5- caffeoylquinic acids, based on the relative intensity of the ions at m/z 191.0567 (quinate ion), 179.0354(60) (caffeate ion) and 173.0464 (dehydrated quinate ion)^26^. Compounds 36, 40 and 44 were dicaffeoylquinic acids (di-CQA), tentatively identified as 3,4-, 1,4- and 4,5-diCQA isomers based on their TOF–MS/MS spectra, which showed the ion at m/z 173.046 as base peak, according to the linkage of a second HCA unit at quinic acid C-4 position. Compound 37 was likely 3,5-diCQA, and compound 49 at m/z 677.1526 was supposed to be a tri-CQA^26^. Chlorogenic acid glycosides were also abundant. The deprotonated molecular ion and theToF-MS2 spectrum of compound 13 were in accordance with 3-CQA dihexoside (Fig. S1). The two sequential losses of 162.05 Da, confirming hexosyl residues occurrence, were observed also for compounds 18 and 24, according to CQA dihexoside isomers. Other tentatively identified HCA-based compounds were hexosyl derivatives of caffeic acid (16,23)^27^, dihydrocaffeic acid (17,19) or p-coumaric acid (15,21), and also two caffeoyl hexosides of the 12-hydroxyjasmonic acids^28^ (38 and 41). Compounds 26, 27 and 29 lacked the dihydroxycinnamate moiety and were tentatively identified as hexosyl 12-hydroxyjasmonic acid (tuberonic acid) isomers.

Seven glycosylated flavonols were tentatively identified, based on the typical neutral losses of common glyconic moieties, and on mass spectrometry features of aglycone ions. The occurrence of some highly glycosylated flavonol derivatives have been previously reported in tomato cvs. commercially available^29^. Quercetin glycosides with different glycosylation degree were identified; specifically, rutin (34), and its hexosyl (30) and pentosyl derivatives (33) derivatives were detected. The deprotonated ion at m/z 771.2039 of compound 30 underwent in the TOF-MS2 experiment a neutral loss of 162.05 Da, giving the ion at m/z 609.1501, which in turn gave rise, through the loss of dehydrated deoxyhexose, to the ions at m/z 463.0904 and 462.0825. Finally, the deprotonated quercetin at m/z 301.0359 was formed following a further loss of a hexose residue. Instead, the fragmentation pattern of compound 33 showed the loss of 132.0448 Da (dehydrated pentose) to give fragment ions that resembled rutin. Compound 43, with the [M-H]^−^ ion at m/z 887.2283 was likely quercetin p-coumaroyl deoxyhexosyl hexosyl pentoside. The neutral loss of 164.049 and 146.0385 Da from the deprotonated molecular ion, generating fragment ions at m/z 723.1937 and 741.1937, providing evidences of the presence of a quercetin linked to a p-coumaroyl moiety. The further loss of 440.1587 Da (likely ascribable to a trisaccharide, formed by a hexose, a deoxyhexose and a pentose residue) from this latter gave rise to the deprotonated flavonol aglycone. Compound 31 was a constitutional isomer of the previous one, differing base on the identity of aglycone, herein the flavonol kaempferol (at m/z 285.0403 in TOF-MS2 spectrum), and dihexosylpentosyl glyconic part. Compounds 35 and 39 were also kaempferol glycosides; the first one lacked a hexose compared to 31; the second was kaempferol rutinoside^23^.

### Mass spectrometric characterization of lipophilic extract constituents

The lipophilic fraction consisted of 13 compounds, mainly fatty acids occurring either in free form (3’, 4’, 6’, 10’-13’) or in glycero-phospholipid structures (5’, 7’–9’) (Table 3), besides two phenylamides (compounds 1’ and 2’), identified as N-feruloyloctopamine and N-feruloyltyramine^30^. These compounds appeared to exert a pivotal role in plant defense, being involved in the response to various stresses, e.g. pathogen infection, wounding, or elicitor treatments^31^.

Compounds 11’ and 12’ were putatively identified as linolenic and linoleic acid, respectively, whereas two trihydroxylated derivatives (3’ and 4’) and one monohydroxylated (6’) occurred at minor retention times, due to their higher polarity. The same elution order was found for C16 fatty acids hydroxy-palmitic (10’) and palmitic (13’). The esterification to the glycerol-phosphate backbone in metabolites 5’, 7’ and 8’ was derived by the presence of common fragment ions at m/z 152.9958 (C_3_H_6_O_5_P^−^) and 96.9696 (H_2_PO_4_^−^), deriving from the loss of the fatty acid residue (Fig. S2). Glycerol-phospholipids in tomato fruits were correlated to physiological adaptations and stress responses^32^. Finally, compound 9’ could be the monomethyl ester of 7’, as suggested by the the product ion at m/z 167.0111 (C_4_H_8_O_5_P^−^).

The Total Ion Chromatogram (TIC) of both hydro-alcoholic and lipophilic samples and MS/MS chromatograms of all assigned compounds were reported in Figs. S3–S20.

### Relative quantitation in response to heavy metal contamination

The relative quantitation of each compound from experimentally contaminated plant samples respect to control ones is reported in Tables S1 and S2. Apart from few metabolites, the observed general trend highlighted a huge increase in the content of both phenols and polyphenols, as well as of amino acids and other polar non-phenolic compounds, in PG-Cd, PG-Pb and PG-Cr samples. On the contrary, N-feruloylamines and less polar compounds, occurring in the lipophilic extracts, decreased when compared to uncontaminated samples. This evidence could be explained as one of the mechanisms that plants use to detoxify themselves from potentially harmful xenobiotic stress^33^. It was found that heavy metals, among which Cd, enhance the production of reactive oxygen species (ROS), which leads to oxidative stress damages^34^. Thus, besides their redox properties, the capacity of polyphenols to act as metal chelators has been proposed as a valuable strategy to keep the amount of free metals in the plant cytoplasm at nontoxic levels^33^.

However, no general conclusion can be drawn about the effect of soil contamination on the phenols overproduction; the plant variety could also have a role in the tolerance to heavy metals. In this context, PR samples showed exactly an opposite behavior respect to PG, so much so that the amount of almost all polar metabolites massively decreased, according to the previous observed significant reduction in ABTS radical cation scavenging capacity^20^. Thus, for PR samples it is reasonable to assume the involvement of defense systems other than the enhancement of biosynthetic routes leading to phenol accumulation (e.g. phenylpropanoid metabolism), including different expression levels of some family genes, or an increased activity of antioxidant enzymes (e.g. peroxidase, catalase, superoxide dismutase)^35^ or an enhanced occurrence of protective pigments, such as carotenoids.

### UV–Vis spectroscopic detection of the main carotenoids

UV–Vis spectroscopic analyses on lipophilic extracts aimed at evaluating the effects of heavy metals contamination on carotenoid pigments. These compounds show a typical absorption between about 400 and 500 nm^36^. As previously reported, some identifying parameters, such as λmax values and the % ratio between the intensity of the band III (at highest nm value) and II (at middle nm value), could be useful for their identification^39^.

**Figure 1:**
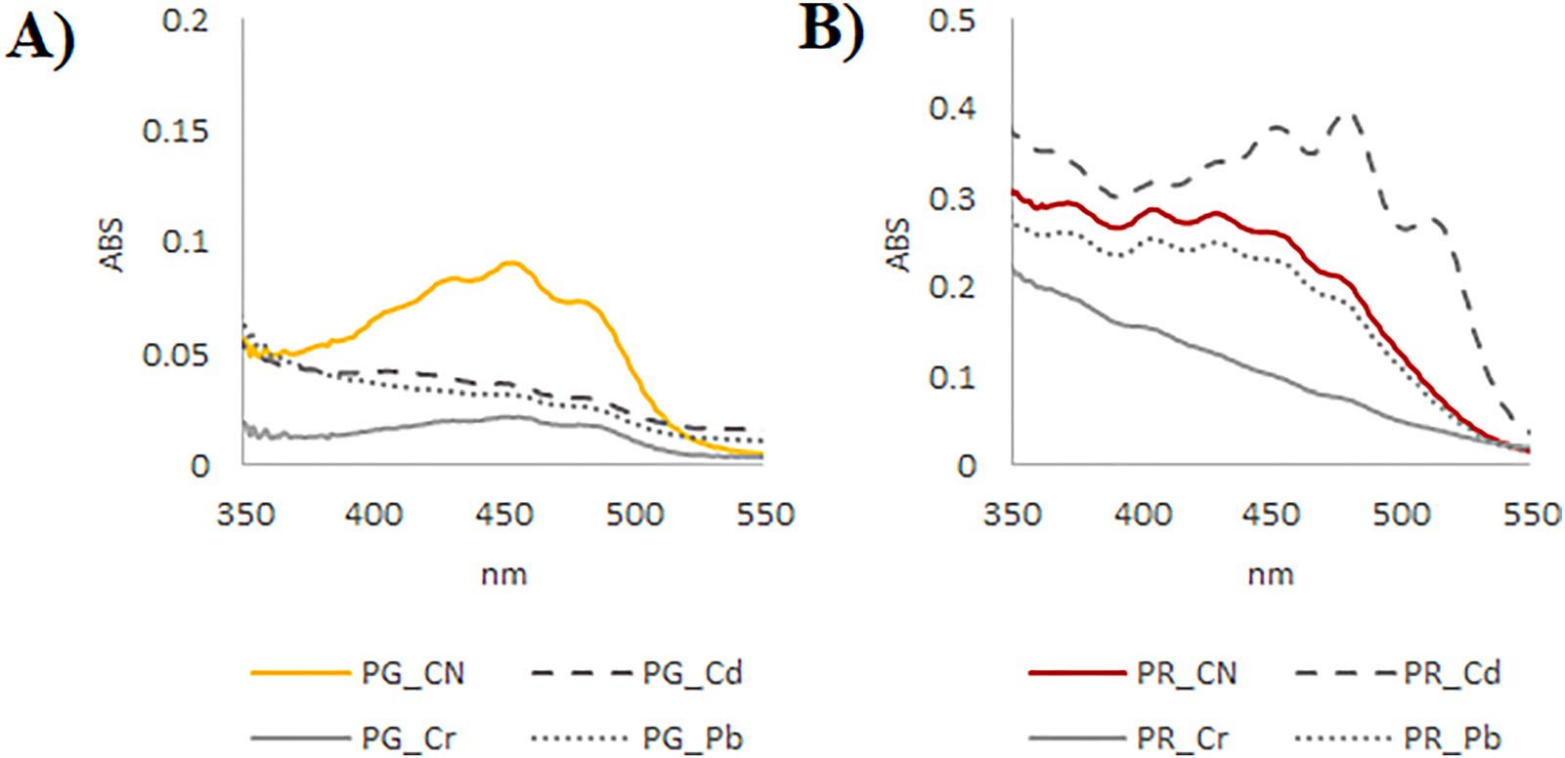
UV–Vis spectra of lipophilic extracts. UV–Vis spectra of lipophilic extracts of PG (**A**) and PR (**B**) samples (CTR = plants grown in uncontaminated soils; Cd, Cr, Pb = plants grown in soils experimentally contaminated by cadmium, chromium and lead). The software used to generate this figure was PeakView—Analyst TF 1.7.

UV–Vis spectrum of PG control sample showed three peaks of maximum absorption at 430, 453 and 478 nm with the middle peak being the most intense one (Fig. 1A). According to previous published data, they could suggest the occurrence of lutein^37^ or β-carotene, as in “Pomodorino vesuviano” tomatoes^38^. However, the % III/II intensity ratio disagreed with those reported therein, suggesting a possible interference of other minor carotenoids in the extract and/or hypochromic effects due to the solvent used.

The exposure to all the considered heavy metals induced a massive decrease in carotenoids’ content in PG samples, as demonstrated by the hypochromic effect that affected the absorbance values at the three characteristic wavelengths. These data correlated with the increase in phenolic compounds and amino acids, as described above, and were in accordance with previous literature data. Indeed, the carotenoid content was found to decrease under Pb stress in tomato plants, whereas total phenol content was observed to enhance, likely due to a plant adaptation to stress that encourage chelation of toxic metal ions in the aerial parts^39^. A similar response was observed as regards carotenoid content after Cd contamination^40^.

Instead, interestingly in PR samples lycopene was identified as the main carotenoid only after Cd contamination (Fig. 1B). In fact, the UV–Vis spectrum was characterized by three maximum absorption peaks at 452, 480 and 512 nm^41^ with the III/II ratio equal to about 70%^37^. Taking into account that among the considered heavy metals, Cd was the only one able to translocate in the tomato fruits of PR plants^20^, it is reasonable to assume a close connection between Cd exposure and lycopene occurrence as plant protection mechanism. Indeed, moderate stress due to environmental factors, which are able to induce ROS production without leading to senescence or necrosis, promotes carotenoid biosynthesis through redox signaling^42^.

### Viability assay

The antiproliferative activity of tomato PG and PR fruit extracts, grown on uncontaminated (PG/PR-CTR) or experimentally contaminated soils by heavy metals (PG/PR-Cd, -Cr and -Pb), was evaluated on gastric and colorectal cancer cells. The results are expressed as IC50 values with 95% confidence limits, and reported in Table 4 for PG and PR. The lowest IC50s represented the highest inhibitory activity, the overlapping confidence limits indicate no significant differences in results.

**Table 4:** Antiproliferative activity of chloroform and methanol extracts of Pomodoro Giallo and San Marzano Cirio 3. Antiproliferative activity expressed as IC50 (mg/L) values, with confidence limits (95% probability, in brackets), of chloroform and methanol extracts of Pomodoro Giallo fruits (PG) from plants grown in uncontaminated (PG-CTR) and contaminated (PG-Cd, PG-Cr, PG-Pb) soils and of San Marzano Cirio 3 (PR) from plants grown in uncontaminated (PR-CTR) and contaminated (PR-Cd, PR-Cr, PR-Pb) soils on AGS, NCI-N87, HT-29 and HCT 116 cells, after 72 h of exposure.

	Pomodoro Giallo			
	AGS	NCI-N87	HT-29	HCT 116
**CHCl**_**3**_**-extract**				
PG-CTR	75.24 (41.56–136.2)	151.5 (121.1–189.5)	52.67 (23.74–116.9)	74.21 (62.73–87.77)
PG-Cd	123.5 (76.32–199.9)	152.2 (122.0–189.9)	62.21 (37.69–102.7)	145.7 (85.80–247.4)
PG-Cr	81.52 (58.02–114.6)	67.89 (61.97–74.38)	29.09 (22.32–37.93)	79.39 (64.66–97.49)
PG-Pb	122.7 (93.91–160.2)	147.3 (115.6–187.8)	78.83 (60.36–102.9)	326.5 (200.8–531.1)
**CH**_**3**_**OH-extract**				
PG-CTR	258.6 (185.7–360.0)	197.3 (173.4–224.4)	204.6 (133.5–313.5)	324.0 (219.1–479.2)
PG-Cd	350.5 (244.8–501.9)	300.0 (169.2–531.9)	283.8 (164.3–490.1)	366.9 (246.5–546.0)
PG-Cr	429.0 (292.3–629.5)	216.8 (163.3–287.8)	234.1 (164.9–334.1)	347.9 (236.9–511.0)
PG-Pb	185.4 (117.8–291.7)	145.4 (129.2–163.7)	144.3 (91.35–227.9)	130.0 (102.8–164.4)

	San Marzano Cirio 3			
	AGS	NCI-N87	HT-29	HCT 116
**CHCl**_**3**_**-extract**				
PR-CTR	55.18 (31.50–96.66)	78.58 (67.69–91.22)	36.78 (22.40–60.40)	70.73 (52.12–95.98)
PR-Cd	102.2 (72.66–143.7)	62.07 (55.70–69.17)	50.81 (40.17–64.27)	98.83 (70.27–139.0)
PR-Cr	95.41 (77.84–116.9)	57.48 (45.59–72.47)	26.98 (23.32–31.31)	80.49 (66.98–96.72)
PR-Pb	122.7 (93.91–160.2)	137.4 (96.84–194.9)	58.82 (36.18–95.64)	97.41 (60.21–157.6)
**CH**_**3**_**OH-extract**				
PR-CTR	158.3 (133.5–187.8)	89.58 (61.53–130.4)	112.7 (89.96–141.1)	314.5 (223.2–443.2)
PR-Cd	695.7 (500.1–967.8)	156.9 (122.9–200.5)	275.8 (158.4–480.3)	266.6 (136.5–520.8)
PR-Cr	200.6 (137.0–293.9)	89.00 (66.82–118.5)	99.31 (64.71–152.4)	193.4 (146.7–255.0)
PR-Pb	156.7 (134.7–182.3)	276.5 (231.0–330.8)	140.8 (95.72–207.0)	183.3 (153.3–219.2)

PG and PR aqueous fractions were not able to inhibit cell viability up to the highest tested concentration (1500 mg/L, data not shown), while the chloroform extracts were the most active samples, with cytotoxic activity higher than methanol extracts. IC50 values of chloroform extracts of PG and PR cultivars grown on uncontaminated soil ranged from 52.67 to 151.5 mg/L and from 36.78 to 78.58 mg/L, respectively, while IC50 values of methanol extracts ranged from 197.3 to 324.0 mg/L for PG and from 89.58 to 314.5 for PR. Extracts from plants grown on contaminated soils did not show statistical differences in antiproliferative activity, except some findings. When tomatoes were from plants irrigated with Cr-solution, both PR and PG chloroform extracts induced cytotoxicity higher than methanol ones on all cell lines except for NCI-N87. A peculiar trend was for PG-Pb chloroform extract with IC50 value higher than that of methanol extract (326.5 vs. 130 mg/L) on HCT-116. PR-Cd-methanol extracts were less cytotoxic than all other metals-methanol extracts when tested on AGS, and less toxic than Cr-methanol extract when tested on HT-29. PR-Pb chloroform extract was also less toxic than all other metals-chloroform extracts on NCI-N87.

No statistically significant differences were found comparing results with those from solvent and negative controls (data not shown).

The EC50 values were chosen as starting point for the subsequent experiments in exposed cells.

### ALDH activity of tomato fruit extracts

**Figure 2:**
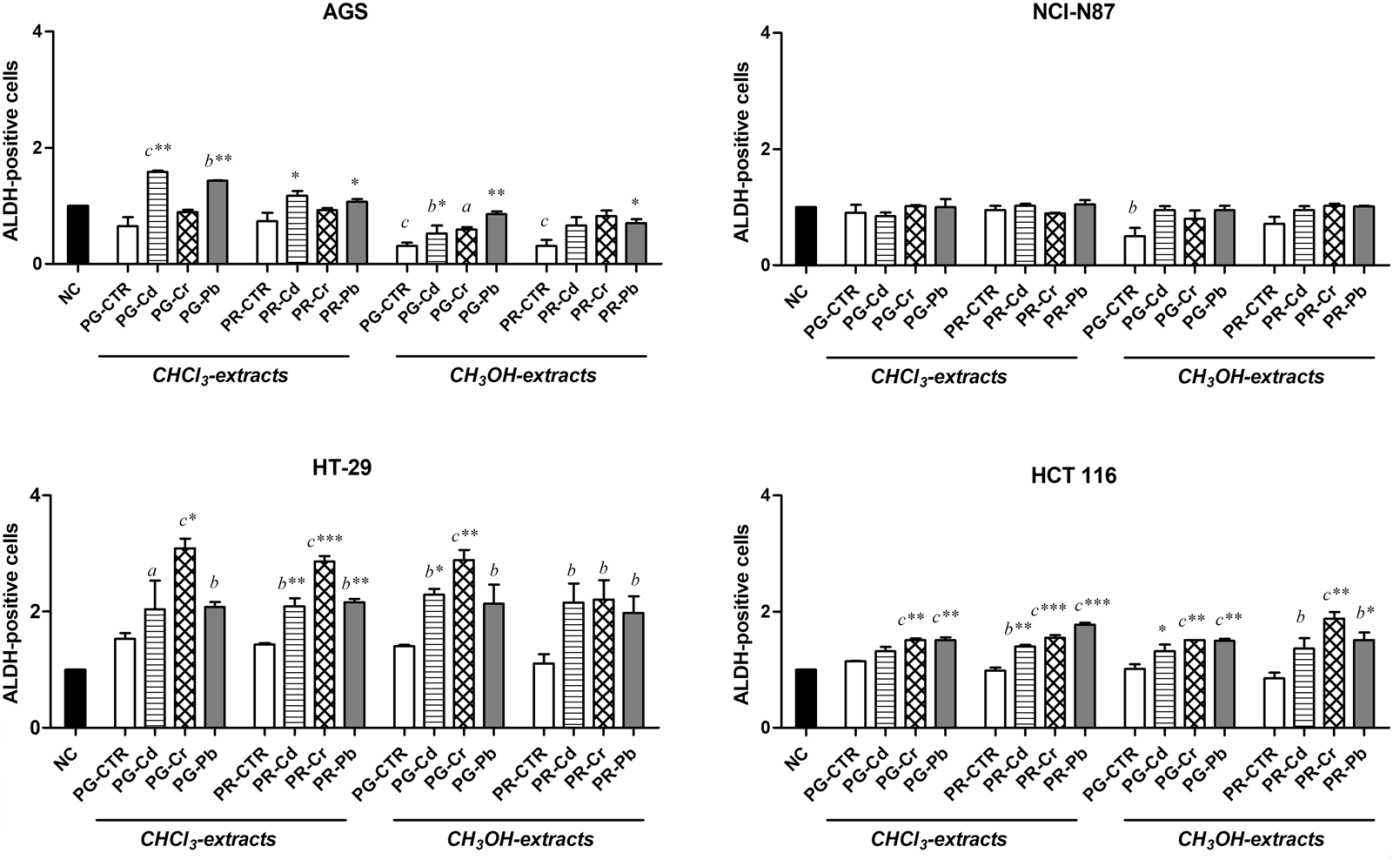
ALDH positive-cell population in AGS, NCI-N87, HT-29 and HCT 116 cells after 72-h. ALDH positive-cell population in AGS, NCI-N87, HT-29 and HCT 116 cells after 72-h treatment with IC50 of chloroform and methanol extracts of Pomodoro Giallo (PG) and San Marzano Cirio 3 (PR) fruits, grown with or without heavy metals (Cd, Cr, Pb) contamination. Results are expressed as means ± standard deviation of three independent experiments. Significant differences from the negative control (NC, untreated cells) are highlighted by letters (**a**: *p* < 0.05; **b**: *p* < 0.01; **c**: *p* < 0.0001—Dunnett’s test). Significant differences between uncontaminated and contaminated tomato extracts are highlighted by asterisks (*p < 0.05; **p < 0.01; ***p < 0.0001—Dunnett’s test). The software used to generate this figure was GraphPad Prism 5.

Tomato extracts from plants grown on uncontaminated soils (PG/PR-CTR) were able to reduce significantly the untreated gastric ALDH-positive cells (NC) (Fig. 2). In detail, the ALDH-positive cells were lower than the observed untreated cells (NC) in methanol extracts of both tomato varieties in AGS, as wells as in PG for NCI-N87. HT-29 and HCT 116 PG/PR-CTR did not show any statistically significant difference compared to NC.

In particular, in AGS the PG-Cd and PG-Pb chloroform extracts showed a statistically significant increase of ALDH-positive cells than both NC and PG-CTR. Moreover, the ALDH activity of Cd-methanol extract was lower than NC, but higher than PG-CTR.

In HT-29, all the extracts statistically increased the ALDH-positive cell population compared to NC, PR chloroform extracts contaminated by heavy metals showed the highest increase of the ALDH activity observed by the remarkable statistically differences compared to the chloroform PR-CTR. In HCT 116, a statistically significant increase of ALDH-positive cells was observed in Cr and Pb methanol fruit extracts compared to both NC and PG/PR-CTR while in NCI-N87 cells, no significant differences were observed. No differences were found between solvent and negative controls (Data not shown).

### Cancer cell cycle arrest

**Figure 3:**
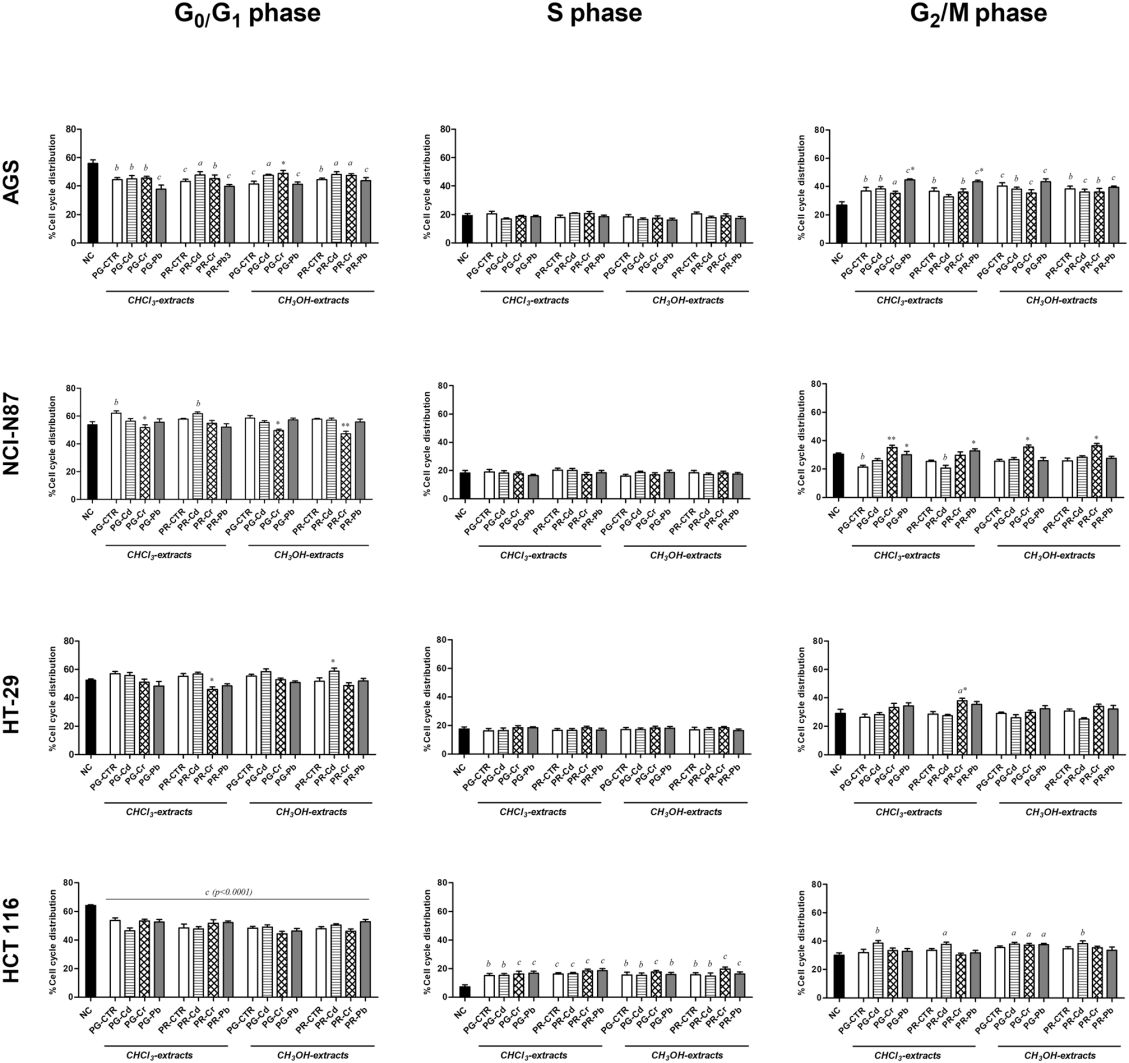
Histograms of AGS, NCI-N87, HT-29 and HCT 116 cell population distribution (in percent) in different cell cycle (G0/G1–S–G2/M) phases after 72-h treatment. Histograms of AGS, NCI-N87, HT-29 and HCT 116 cell population distribution (in percent) in different cell cycle (G0/G1–S–G2/M) phases after 72-h treatment with IC50 of cloroform and methanol extracts of Pomodoro Giallo (PG) and San Marzano Cirio 3 (PR) fruits, grown with or without heavy metals (Cd, Cr, Pb) contamination. Results are expressed as means ± SD of three separate experiments. Significant differences from the negative control (NC, untreated cells) are highlighted by letters (**a**: *p* < 0.05; **b**: *p* < 0.01; **c**: *p* < 0.0001—Dunnett’s test). Significant differences between uncontaminated and contaminated tomato extracts are highlighted by asterisks (*p < 0.05; **p < 0.01; ***p < 0.0001—Dunnett’s test). The software used to generate this figure was GraphPad Prism 5.

In AGS (Fig. 3), samples showed a significant decrease of the distribution of cells during the G0/G1 phase as well as a significant increase during G2/M phase compared to NC, suggesting that tomato extracts were able to induce G2-arrest. No variations were observed during S phase.

In NCI-N87, only the PG-CTR chloroform extract was able to block cell cycle in G1 phase since a remarkable accumulation of the cells in the G0/G1 phase and their significant decrease in G2/M phase compared to NC were observed.

Moreover, the NCI-N87 cells, after 72-h treatment with the chloroform extract of PG and the methanol extracts of both tomato cultivars contaminated by Cr, statistically decreased in G0/G1 cell population. Furthermore, the percentage of cell distribution was significantly different between PG/PR-Cr methanol extracts and their PG/PR controls during the G2/M phase. Finally, the chloroform extracts of both tomato cultivars grown in soils contaminated by Pb were able to induce a statistically significant increase in the fraction of NCI-N87 cells in the G2/M phase. Also in NCI-N87 cells, no variations were observed during S phase. Thus, both gastric cell lines showed the cycle arrest in G2/M phase.

Regarding HT-29, the percentage of cell cycle distribution significantly decreased during G0/G1 phase and increased during G2/M phase (compared to PR-CTR) when cells were exposed to PR-Cr chloroform extracts. No variations were observed during S phase.

No significant differences were found between solvent and negative controls (Data not shown).

### Apoptotic and/or necrotic effects

**Figure 4:**
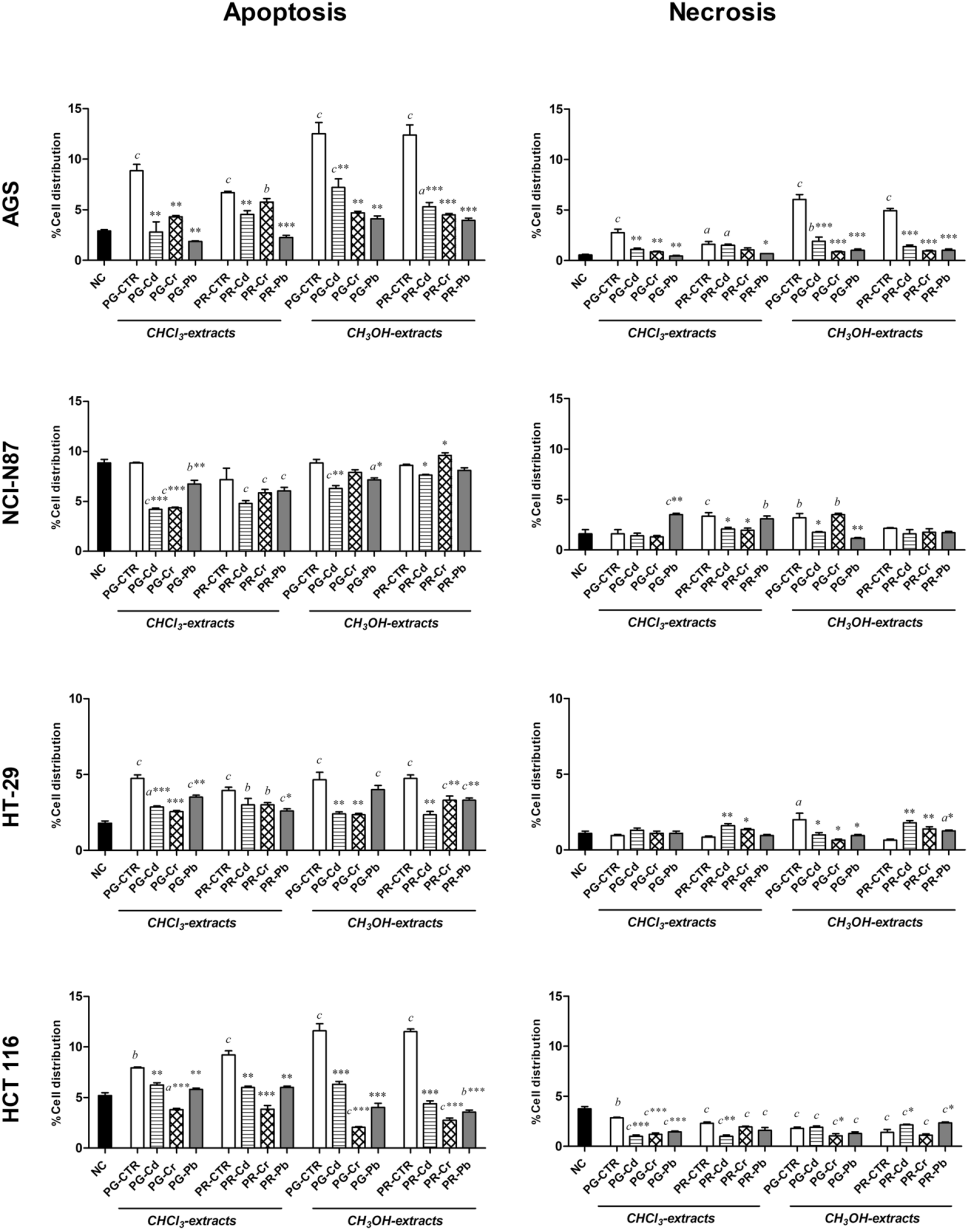
Histograms of apoptotic and necrotic AGS, NCI-N87, HT-29 and HCT 116 cell distribution (in percent) after 72-h treatment. Histograms of apoptotic and necrotic AGS, NCI-N87, HT-29 and HCT 116 cell distribution (in percent) after 72-h treatment with IC50 of chloroform and methanol extracts of Pomodoro Giallo (PG) and San Marzano Cirio 3 (PR) fruits, grown with or without heavy metals (Cd, Cr, Pb) contamination. Results are expressed as means ± SD of three separate experiments. Significant differences from the negative control (NC, untreated cells) are highlighted by letters (**a**: *p* < 0.05; **b**: *p* < 0.01; **c**: *p* < 0.0001—Dunnett’s test). Significant differences between uncontaminated and contaminated tomato extracts are highlighted by asterisks (*p < 0.05; **p < 0.01; ***p < 0.0001—Dunnett’s test). The software used to generate this figure was GraphPad Prism 5.

Cell apoptosis and necrosis data are in Fig. 4.

AGS cells treated with PG-PR-CTRs had a significant increase in apoptotic and necrotic cells compared to NC. No cell apoptosis was observed in NCI-N87 exposing them to PG/PR-CTR chloroform and methanol extracts as no statistical differences from NC were observed; however, a significant increase of cell necrosis was highlighted testing PR-CTR-chloroform and PG-CTR-methanol extracts. Furthermore, a significant increase of apoptotic cells was found exposing HT-29 cells to PR/PG-CTR extracts, while a significant increase of necrotic cells was obtained with PG-CTR methanol extract (*p* < 0.05 from NC). Finally, PG/PR- CTR extracts were able to cause a statistically increase of the apoptotic cell population and a decrease of the necrotic cell population in HCT-116 (compared to NCs).

As regards the extracts of plants grown in contaminated soils, we observed a remarkable apoptosis (statistically significant increase of cell distribution percentage of cells treated than NC) in AGS cells, after 72-h treatment with the PG/PR- Cd methanol extracts and PR-Cr chloroform extract, and in HT-29 cells, after 72-h treatment with PG/PR-Pb chloroform and methanol extracts (*p* < 0.0001 from NC), PG-Cd chloroform extracts and with PR-Pb chloroform and methanol extracts. However, in all cancer cell lines, a significant reduction of apoptosis was evident comparing the percentage of distribution of the cells exposed to Cd, Cr and Pb-extracts to that of the cells exposed to PG/PR-CTRs.

Regarding the necrotic effect, a remarkable necrosis was observed in AGS cells, after 72-h treatment with PR-Cd chloroform and PG-Cd methanol extracts, in NCI-N87 cell line, after the treatment with the PG/PR- Pb chloroform extracts and the PG-Cr methanol extract, and in HT-29, after treatment with PR-Pb methanol extract. Differently, in HCT 116, soil contamination with Cd, Cr and Pb significantly reduced the ability of tomato fruit extracts to induce necrosis in all cancer cell lines compared to the corresponding NCs.

No statistically significant differences were found between solvent and negative controls (Data not shown).

## Discussion

Pomodoro Giallo e San Marzano Cirio 3 extracts from plants grown in uncontaminated or experimentally contaminated soils were studied in order to unravel as heavy metals influence tomato secondary metabolites, and their cytotoxicity, when concentrated in the extract mixture form, towards cancer stem-like cells.

The starting point of our experimental design was the evaluation of the antiproliferative activity of tomato extracts to obtain the IC50 values to use in the subsequent tests. The results showed that the PG and PR chloroform extracts from plants grown on uncontaminated soil, exerted the highest cytotoxic activity, in line with previous data which demonstrated that lipophilic extracts of two tomato varieties inhibited the growth of gastric cancer cells^43^. In addition, a high cytotoxicity on hepatocarcinoma cells exerted by tomatoes, due to carotenoids detected in lipophilic extracts of San Marzano Cirio 3 (lycopene and carotene) and of Pomodoro Giallo (carotene) was observed^1^. Furthermore, previous findings reported that lycopene could affect CSC self-renewal by inhibition of Wnt/β-catenin signaling^16^, an important pathway modulating cell proliferation, migration, apoptosis, differentiation and stem cell self-renewal^44,45^, while β-carotene was found to inhibit self-renewal characteristics of CSCs in neuroblastoma cells, reducing the expression of several stem cell markers^19^.

Meanwhile, in HCT 116, no statistically differences were observed in cell cycle analysis between extracts from tomatoes of plants grown in contaminated and uncontaminated soil. However, all the extracts were able to induce a remarkable decrease in the G0/G1 cell population together with evident increases in S and G2/M cell populations compared to NC, suggesting that tomatoes extracts could alter the S transition and G2/M phase in cells Cd-treated.

Cytotoxicity data of extracts from PG and PR plants were grown in experimentally contaminated soil were peculiar. PG-Cr chloroform extract induced an increase in inhibiting the viability of all cell lines tested, while PR-Cd methanol extract determined a cytotoxicity reduction of AGS and HT-29 cells. In this context, it was found that Cr causes free ROS overproduction^46–48^, which affects cell differentiation, proliferation and survival as well as the cell redox homeostasis^49,50^, and that Cr-induced ROS altered the enzymatic antioxidant system with genotoxic, ultrastructural, and photosynthetic changes in plants^51^. Furthermore, a protective role of polyphenols against Cd-induced cytotoxicity was suggested^52,53^, while an enrichment in polyphenols in PR methanol extracts, also from Cd-treated soils, reduced cytotoxicity.

HCT-116 cells viability decreased after treatment with PG-Pb and PR-Pb methanol extracts. It was reported that Pb was not able to translocate from roots to fruits^20^, concentrating at leaf level at doses equal to 2.2 and 0.9 mg/Kg dry weigh for PG and PR, respectively (Table S4). Nevertheless, it was found a correlation between Pb content in the soils and the alteration of edible vegetables characteristics such as the ROS production as defense strategy^54^.

Considering the antiproliferative activity results as the starting point for all further assays performed in this study, it was deemed useful summarising outcomes of ALDH reduction, cycle arrest, apoptosis and necrosis assays to facilitate the reader in the general understanding of the observed activities.

Thus, the differences in biological activities between selected cancer cells treated with tomatoes from plants grown in uncontaminated (PG/PR-CTR) or contaminated soil (PG/PR-Cd, Cr, Pb) to untreated cells (NC), were expressed in grayscale and reported in the chromatic Scheme S1.

No significant differences were observed between PG- and PR-CTRs chloroform extracts and respective NCs underlining that no variations in ALDH reduction occurred. Differently, treating AGS cells with PG-Cd/Pb, HT-29 with PG- and PR-Cd/Cr/Pb and HCT116 with PG- and PR-Cr/Pb and PR-Cd chloroform extracts, ALDH reduction decreased (light gray), with a relative increase of the cellular aberrant phenotype, mostly of the colorectal cancer cells. Shin and collaborators^55^ explained that under high oxygen tension conditions (for example, the increase in ROS potentially caused by metals), β-carotene showed tumor-promoting effects. A similar trend was observed for methanol extracts except in the treatment of AGS cells with PG- and PR-CTR and PG-Cd/Cr and NCI-N87 with PG-CTR where an evident increase in ALDH reduction (dark grey) was observed showing a surprising reduction of the aberrant phenotype in gastric cancer cells. Surely, considering ALDH as stem cell marker^56^, we may assert that PG- and PR-CTR methanol extracts, reached in polyphenols (especially, quercetin) and hydroxycinnamic acids^1,57^, play a key role in reducing gastric cancer stem-like cells aberrant phenotype. Furthermore, Zhou et al.^58^ demonstrated that quercetin was able to inhibit in vivo proliferation, angiogenesis, cancer stem cell-ALDH1 marker expression in prostate cancer cells. It should be noted that the polyphenol content found by Piscitelli et al.^1^ in PG methanol extracts (474 mmol eq. quercetin) was about nine times higher than the polyphenol content found in PR (51.5 mmol eq. quercetin), explaining the more evident ALDH reduction activity exerted by PG than PR.

On the other hand, it is not negligible that treating PG plants with Cd and Cr, secondary metabolites potentially increased in fruits as a reaction to the ROS productions as shown by many studies which reported the increase in polyphenol content in vegetables exposed to heavy metals including Cd and Cr^59–61^.

As regards cell cycle arrest, although the expression of proteins involved in the cell cycle was not evaluated in this study, the distribution of the cells in the different phases of the cycle is useful to understand if the cell arrest occurred or not. Hence, the cycle arrest occurred especially when PG and PR (of both untreated and treated plants) were tested on AGS gastric cells (dark grey). In a recent study, Barone and collaborators^43^ demonstrated that the lipophilic extract of Corbarino tomato variety was able to arrest gastric cell proliferation in G1 phase. Notably, in HT-29 colorectal cells, an evident cycle arrest was observed testing PG methanol extracts of tomatoes from plants grown in contaminated soil, suggesting, once again, a potential correlation between heavy metal contamination and increase in polyphenols content^59–61^. Surely, AGS, HT-29 and HCT-116 cells were also very sensitive to apoptosis when tested with PG- and PR-CTR extracts, demonstrating that tomatoes are able to induce apoptosis in cancer cells. Moreover, a downregulation of Bcl-2 in HT-29 after tomato digestates attributable mainly to lycopene was found^62,63^. In this study, when HT-29 cells were treated with chloroform extracts rich in carotenoids, apoptosis was induced also when these tomato extracts were from plants grown with heavy metals. Furthermore, in previous studies^64,65^ tomato-based food product induced apoptosis in colon cancer HT-29 cell lines and in primary human prostate cancer cells. Undoubtedly, tomato extracts induced apoptosis more than necrosis in cancer cells but tomato extracts from plants grown in uncontaminated soil induced also necrosis in AGS cells (dark grey), resulting again as the most sensitive cells. Differently, the least sensitive cells (light grey) to apoptosis and necrosis were NCI-N87 and HCT-116; in NCI-N87 especially when they were treated with PG extracts from contaminated plants while in HCT-116, necrosis was inhibited both with PG- and PR-CTRs or PG- and PR-heavy metals.

## Methods

All methods were carried out in accordance with relevant guidelines and regulations.

### Sampling and extraction

Tomato plants of two Italian varieties (Pomodoro Giallo, PG and San Marzano Cirio 3, PR) were grown in soils uncontaminated, and experimentally contaminated by cadmium (Cd), chromium (Cr) or lead (Pb)^20^ (Tables S3 and S4). Cr and Pb were selected because of their occurrence in Agro Nocerino Sarnese crop soil at concentrations respectively equal to 198.4 ± 31.9 and up to 215 mg/kg dry weight^66,67^ higher than the maximum limits permitted by Italian law (Legislative Decree 152/2006 equal to150 and 100 mg/kg dry weight, respectively for Cr and Pb); while Cd was chosen for its known ability to translocate in tomato fruits^67^. The experimental concentrations used to contaminate the soils (Table S3), were extremely high, far from those found in uncontaminated soils, in order to simulate plant growth in an extremely polluted environment as suggested by Buondonno et al.^68^ and reported by Piscitelli et al.^20^. The concentrations (mg/Kg dry weight) of Cr, Cd and Pb, in bulk soil, roots, leaves and fruits of PG and PR have been detected in our previous work^20^ (Table S4).

After harvesting, tomato fruits were instantly frozen in dry ice and stored at − 80 °C.

About 2 g of frozen samples were lyophilized (− 150 mTorr, − 80 °C, 96 h) in a freeze-dryer, and pulverized before extraction. The fruit solid/liquid extraction was conducted by using a biphasic solution (chloroform, methanol and water, 2:1:1). The separation of the two immiscible liquid phases was performed by discontinuous liquid/liquid extraction and, as a consequence, a hydroalcoholic and a lipophilic extract were obtained (Fig. S21). These latter were chemically profiled through UHPLC-ESI-QqTOF-MS/MS and UV–Vis spectroscopy. Based on their chemical composition, the hydroalcoholic extracts were further partitioned into aqueous and methanol fractions by means of Sep-pakC18 cartridges. For the cytotoxicity assays, the chloroform extracts were dissolved in DMSO (2%), and aqueous, and methanol fractions in water and methanol (5%), respectively. The stock solutions were appropriately diluted to reach no toxic solvents percentages for cells in test dilutions (< 1% for methanol, < 0. 1% for DMSO).

### Chemical composition analyses

A Shimadzu NEXERA UHPLC system was used with a Luna Omega C18 column (1.6 μm particle size, 50 × 2.1 mm i.d., Phenomenex, Torrance, CA, USA). Chromatographic separation was optimized with a linear elution gradient of water (A) and acetonitrile (B), both with 0.1% formic acid, as it follows. For hydroalcoholic extracts the gradient started at 10% B, kept for 0.75 min, and linearly ramped to 45% B in 5 min, then to 95% in other 2.5 min and maintained for 1 min, before restoring the initial conditions. For lipophilic extracts the gradient started at 20% B and ramped at 98% B in 26 min, and then after an isocratic step of 2 min the initial mobile phase composition was restored. In both cases, the flow rate was 0.5 mL min^−1^ with an injection volume of 2.0 μL. The AB SCIEX TripleTOF 4600 hybrid system (AB Sciex, Concord, ON, Canada) with a DuoSpray ion source operating in negative electrospray ionization was coupled to UHPLC instrument for HRMS analyses. The APCI probe of the source was used for fully automatic mass calibration using the Calibrant Delivery System. In Table S5 mass spectrometry parameters optimized for both fractions are listed. Data were collected by information dependent acquisition using a TOF–MS survey scan of 100–1500 Da (100 ms accumulation time) and eight dependent TOF–MS/MS scans of 80–1500 Da (100 ms accumulation time). Data processing was performed using the PeakView—Analyst TF 1.7 software. Relative quantitation was performed by using a SCIEX Triple Quadrupole 3500 Instrument (AB Sciex, Concord, ON, Canada) coupled to a HPLC 1260 INFINITY II system (Agilent, Santa Clara, CA, USA) and operating in SIM (Selected Ion Monitoring) data acquisition mode, using the same potentials listed above.

Lipophilic tomato extracts also underwent UV–Vis spectroscopic analysis for carotenoids detection. Thus, samples were dissolved in dichloromethane (200 ppm, final concentration) and spectral data were acquired by the Cary 100 spectrophotometer (Agilent, Milano, Italia) in the range 200–800 nm.

### Cell cultures

Gastric adenocarcinoma (AGS; ATCC CRL-1739) and carcinoma (NCI-N87; ATCC CRL-5822) cells, colorectal adenocarcinoma (HT-29; ATCC HTB-38) and carcinoma (HCT-116; ATCC CCL-247) cells were purchased from ATCC (Manassas, VA, USA). AGS cells were maintained in F-12K medium (ATCC), NCI-N87 were maintained in RPMI-1640 medium, HT-29 and HCT 116 cells were maintained in McCoy’s 5A modified medium. All the culture media were complexed with 10% fetal bovine serum (ATCC). All the cells tested were placed at 37 °C in a humidified atmosphere of 5% CO_2_ to retain the proliferating condition.

### Viability assay

Cell proliferation was determined using CellTiter-Glo Luminescent assay following the manufacturer’s guidelines (Promega, Alexandria, NSW, Australia). Briefly, cells (1 × 10^4^ cells/well) were plated in black flat-bottomed 96-well plates in the culture medium for 24 h. Then, test solution (10 to 1500 mg/L for aqueous and methanol extracts; 10 to 500 mg/L for chloroform extracts) were prepared using the cell culture medium at different concentrations chosen after range finding tests.

Each plate had negative control (NC) as well as solvent controls.

After an incubation of 72 h, CellTiter-Glo reagent (10 μL) was added, and plates were mixed gently for 2 min on an orbital shaker to induce cell lysis. Finally, the plates were incubated at room temperature for 10 min to stabilize luminescent signal and read at a luminometer.

The results, obtained from three independent tests, were expressed as the concentration inhibiting the 50% cell growth rate (IC50), calculated as 100—cell viability rate. The cell viability rate was calculated with the following formula: (extract absorbance—control absorbance)/control absorbance × 100. The IC50s here obtained were used to treat cells in the subsequent tests.

### Aldehyde dehydrogenase (ALDH) activity assay

ALDH activity assay was performed using the ALDEFLOUR™ kit following the manufacturer’s guidelines (StemCell Technologies, Canada). Briefly, 1 × 10^6^ cells/mL were treated for 72 h with the IC50s of tomato extracts, then resuspended in ALDEFLUOR buffer in test tubes and 5 µL of the activated BAAA was added. Then, an aliquot of 500 µL of the suspension was transferred to the blank tubes with 5 µL ALDH inhibitor diethylaminobenzaldehyde (DEAB, 1.5 nM). Untreated cells were used as negative control (NC). Solvent controls were also prepared. After incubation at 37 °C for 45 min, the tubes were centrifuged for 5 min at 1000 rpm and the supernatant was removed. The cells of test tube were re-suspended in 500 µL of Aldefluor Assay Buffer. ALDHbr cells were analyzed with FACS (Accuri C6 Flow Cytometer, Accuri).

### Cell cycle analysis

The cell cycle analysis was conducted following the manufacturer’s guidelines (Abcam, Cambridge, UK). After 72 h of incubation with the IC50s of tomato extracts, 1 × 10^6^ cells were washed with PBS, fixed in cold ethanol 70% (v/v) and stored at 4 °C at least for 2 h. Then, cells were centrifuged at 1000 rpm, washed twice with PBS, and re-suspended in 200 µL of propidium iodide (PI, 50 µg/mL) and RNase staining solution (0.2 mg/mL). Cells not treated with tomato extracts were used as negative control (NC). The stained samples were incubated 30 min at 37 °C and analyzed by using an Accuri C6 Flow Cytometer. Solvent controls were included.

### The Annexin V-FITC assay

Annexin V–fluorescein isothiocyanate (FITC) assay was performed using the manufacturer’s guidelines (Annexin V-FITC Apoptosis Detection Kit, Abcam) to detect apoptotic and necrotic cells. Briefly, 1 × 10^6^ cells were seeded in a 6-well plate and treated with the IC50s of tomato extracts for 72 h. The adherent cells were washed twice with cold PBS, re-suspended in binding buffer and stained using Annexin V-FITC/PI. Cells not treated with tomato extracts were used as negative control (NC). Solvent controls were included. All samples were analyzed by Accuri C6 Flow Cytometer using Annexin V FITC signal detector (usually FL1) and PI signal detector (usually FL2) to assess the percentage of intact and viable cells (Annexin-V and PI-negative signal), apoptotic (Annexin-V positive and PI-negative signal) and necrotic cells (Annexin-V and PI-positive signal).

### Statistical analysis

Viability assay results were expressed as IC50 values, from three independent experiments, and were obtained by GraphPad Prism 5 analysis (Inc., CA, USA) using non-linear regression (log agonist vs. normalized response-variable slope) with 95% confidence range. All data from ALDH activity, cell cycle analysis and Annexin V-FITC assays were expressed as the mean values from three independent experiments with their standard deviation. Statistical differences were calculated by One-way ANOVA and Dunnett’s multiple comparison test using GraphPad Prism. Differences were considered as significant when the *p* values were < 0.05.

## Conclusions

In light of the above, the Sarno River basin is a very fertile area but it is also very compromised by anthropogenic activities and it is necessary to explore any possible solution to avoid that polluting sources affect food and then general public health. Herein, it was showed that heavy metals (Cd, Cr and Pb) contamination could cause cellular stress, also affecting the diverse activation of secondary metabolism pathways as defense strategy. This leads to a different content of polyphenol and carotenoid constituents, so much so that contrasting effects on cancer cells were observed based on tomato variety, the extract polarity, heavy metal identity, and tested cell line. Different cellular activities could be due to a broad spectrum of adaptive responses with a possible hormetic effects in cancer cells. This study also suggests that the 72-h treatment with tomato fruit extracts harvested in soils contaminated by heavy metals interferes with the ALDH activity of cancer cell studied. It should be emphasized that among the three heavy metals studied, Cd translocates from roots to fruits and although soil contaminated by heavy metals induces tomato plant towards a modulation of secondary metabolites production as strategy to contrast the contamination, it is to consider the potential adverse health effect due to Cd in the fruits.

## Supplementary Information

Supplementary Information.

## Data Availability

All authors confirm that all data generated or analysed during this study are included in this published article and its supplementary information files.
