# Supplementary material for: Cytotoxic evaluation and chemical investigation of tomatoes from plants (Solanum lycopersicum L.) grown in uncontaminated and experimentally contaminated soils
Source: Sci Rep. 2022 Jul 29;12:13024. doi: 10.1038/s41598-022-13876-w (PMC9338037; doi:10.1038/s41598-022-13876-w)
Supplement: Supplementary file 1 — Supplementary Information. [file 41598_2022_13876_MOESM1_ESM.docx]

**Cytotoxic evaluation and chemical investigation of tomatoes from plants (Solanum lycopersicum L.) grown in uncontaminated and experimentally contaminated soils**

Chiara Russo^a^, Daniela Barone^b^, Margherita Lavorgna^a^*, Concetta Piscitelli^a^, Marcella Macaluso^c^, Severina Pacifico^a^, Simona Piccolella^a^, Antonio Giordano^c^, Marina Isidori^a^

^a^ Dipartimento di Scienze e Tecnologie Ambientali, Biologiche e Farmaceutiche, Università della Campania “Luigi Vanvitelli”, Via Vivaldi 43, 81100 Caserta, Italy

^b^ Cell Biology and Biotherapy Unit, Istituto Nazionale Tumori ‑ IRCCS ‑ Fondazione G. Pascale, 80131 Napoli, Italy.

^c^ Sbarro Institute for Cancer Research and Molecular Medicine, Department of Biology, College of Science and Technology, Temple University, Philadelphia, PA, USA

*Corresponding authors:

Margherita Lavorgna

Email: [margherita.lavorgna@unicampania.it](mailto:margherita.lavorgna@unicampania.it)

**Supporting Information**

**Table S1.** Relative amount of each compound in hydro-alcoholic extracts under study, compared with control sample. Increase % is highlighted by black dots (■ 1-33%; ■ 34-66%; ■ 67-100%; ■ > 100%), whereas decrease % is highlighted by white dots (■ 1-33%; ■ 34-66%; ■ 67-100%). n.q. = not quantifiable

| **Peak n.** | **Tentative assignment** | **PG-Cd** | **PG-Pb** | **PG-Cr** | **PR-Cd** | **PR-Pb** | **PR-Cr** |
| --- | --- | --- | --- | --- | --- | --- | --- |
| **1** | Aspartic acid |  |  |  |  |  |  |
| **2** | Glutamic acid |  |  |  |  |  |  |
| **3** | Citric acid |  |  |  |  |  |  |
| **4** | Methylcitric acid |  |  |  |  |  |  |
| **5** | 4-hydroxyphenyl β-sophoroside |  |  |  |  |  |  |
| **6** | Phenylalanine |  |  |  |  |  |  |
| **7** | Hexosyl phenylalanine |  |  |  |  |  |  |
| **8** | Hydroxybenzoic acid hexoside |  |  |  |  |  |  |
| **9** | Tryptophan |  |  |  |  |  |  |
| **10** | Leucinopine |  |  |  |  |  |  |
| **11** | Pantothenic acid 4’-*O*-glucoside |  |  |  |  |  |  |
| **12** | 3-CQA |  |  |  |  |  |  |
| **13** | 3-CQA dihexoside |  |  |  |  |  |  |
| **14** | Unknown |  |  |  |  |  |  |
| **15** | *p*-coumaric acid hexoside 1 |  |  |  |  |  |  |
| **16** | Caffeic acid hexoside 1 |  |  |  |  |  |  |
| **17** | Dihydrocaffeic acid hexoside 1 | n.q. | n.q. | n.q. | n.q. | n.q. | n.q. |
| **18** | 3-CQA hexoside 1 | n.q. | n.q. | n.q. | n.q. | n.q. | n.q. |
| **19** | Dihydrocaffeic acid hexoside 2 |  |  |  |  |  |  |
| **20** | *N*-phenylacetyl aspartic acid |  |  |  |  |  |  |
| **21** | *p*-coumaric acid hexoside 2 |  |  |  |  |  |  |
| **22** | Unknown |  |  |  |  |  |  |
| **23** | Caffeic acid hexoside 2 |  |  |  |  |  |  |
| **24** | 3-CQA hexoside 2 |  |  |  |  |  |  |
| **25** | 4-CQA |  |  |  |  |  |  |
| **26** | Tuberonic acid hexoside 1 |  |  |  |  |  |  |
| **27** | Tuberonic acid hexoside 2 | n.q. | n.q. | n.q. | n.q. | n.q. | n.q. |
| **28** | 5-CQA |  |  |  |  |  |  |
| **29** | Tuberonic acid hexoside 3 |  |  |  |  |  |  |
| **30** | Quercetin dihexosyl deoxyhexoside |  |  |  |  |  |  |
| **31** | Kaempferol dihexosyl pentosyl deoxyhexoside |  |  |  |  |  |  |
| **32** | 8-hydroxy-2,7-dimethylocta-4,6-dienoic acid hexoside |  |  |  |  |  |  |
| **33** | Quercetin pentosyl rutinoside |  |  |  |  |  |  |
| **34** | Rutin |  |  |  |  |  |  |
| **35** | Kaempferol pentosyl rutinoside |  |  |  |  |  |  |
| **36** | 3,4-diCQA |  |  |  |  |  |  |
| **37** | 3,5-diCQA |  |  |  |  |  |  |
| **38** | 12-*O*-(caffeoylhexosyl) jasmonate 1 |  |  |  |  |  |  |
| **39** | Kaempferol rutinoside |  |  |  |  |  |  |
| **40** | 1,4-diCQA |  |  |  |  |  |  |
| **41** | 12-*O*-(caffeoylhexosyl) jasmonate 2 | n.q. | n.q. | n.q. |  |  |  |
| **42** | Caffeic acid derivative |  |  |  |  |  |  |
| **43** | Quercetin *p*-coumaroyl deoxyhexosyl hexosyl pentoside |  |  |  |  |  |  |
| **44** | 4,5-diCQA | n.q. | n.q. | n.q. | n.q. | n.q. | n.q. |
| **45** | Caffeic acid derivative |  |  |  |  |  |  |
| **46** | Dihydrocaffeic acid derivative | n.q. | n.q. | n.q. |  |  |  |
| **47** | *p*-coumaric acid derivative | n.q. | n.q. | n.q. |  |  |  |
| **48** | *p*-coumaric acid derivative | n.q. | n.q. | n.q. |  |  |  |
| **49** | Tri-CQA |  |  |  |  |  |  |

**Table S2.** Relative amount of each compound in lipophilic extracts under study, compared with control sample. Increase % is highlighted by black dots (■ 1-33%; ■ 34-66%; ■ 67-100%; ■ > 100%), whereas decrease % is highlighted by white dots (■ 1-33%; ■ 34-66%; ■ 67-100%). n.q. = not quantifiable

| **Peak n.** | **Tentative assignment** | **PG-Cd** | **PG-Pb** | **PG-Cr** | **PR-Cd** | **PR-Pb** | **PR-Cr** |
| --- | --- | --- | --- | --- | --- | --- | --- |
| **1’** | *N*-feruloyloctopamine |  |  |  |  |  |  |
| **2’** | *N*-feruloyltyramine |  |  |  |  |  |  |
| **3’** | Trihydroxy-octadecenoic acid |  |  |  |  |  |  |
| **4’** | Trihydroxy-octadecadienoic acid |  |  |  |  |  |  |
| **5’** | Linolenoyl-glycerol-3-phosphate |  |  |  | n.q. | n.q. | n.q. |
| **6’** | Hydroxy-linoleic acid |  | n.q. |  |  |  |  |
| **7’** | Linoleoyl-glycerol 3-phosphate |  |  |  |  |  |  |
| **8’** | Palmitoyl-glycerol 3-phosphate |  |  |  | n.q. | n.q. | n.q. |
| **9’** | Linoleoyl-lysophosphatidic acid monomethyl ester |  |  |  |  |  |  |
| **10’** | Hydroxy-palmitic acid |  |  |  |  |  |  |
| **11’** | Linolenic acid |  |  |  |  |  |  |
| **12’** | Linoleic acid |  |  |  |  |  |  |
| **13’** | Palmitic acid |  |  |  |  |  |  |

**Table S3.** Amounts (expressed in g) of CdCl_2_ (as source of Cd), Cr_4_(SO_4_)_5_(HO)_2_ (as source of Cr) and Pb(NO_3_)_2_ (as source of Pb) added to the experimental soils to have Cd, Cr and Pb at 10%, in relation to soil CEC (Piscitelli et al., 2020)

|  | NC | Cd | Cr | Pb |
| --- | --- | --- | --- | --- |
| CdCl_2_ | - | 3.1 | - | - |
| Cr_4_(SO_4_)_5_(HO)_2_ | - | - | 2.1 | - |
| Pb(NO_3_)_2_ | - | - | - | 5.7 |
| KNO_3_ | 3.4 | 3.4 | 3.4 | - |
| K_2_SO_4_ | 2.5 | 2.5 | - | 2.5 |

**.**

**Table S4.** Concentrations (mg/Kg dry weight) of Cr, Cd and Pb, detected by ICP-OES analysis, in bulk soil, roots, stems, leaves and fruits of PG and PR

Concentrations (mg/Kg dry weight) of Cr, Cd and Pb, detected by ICP-OES analysis, in bulk soil, roots, stems, leaves and fruits of PG and PR. Results are reported as mean values (3 independent experiments) ± Standard Error. Significant differences between PG and PR for each part and level% CEC of Cr, Cd and Pb were analysed by 2way-ANOVA, Bonferroni for *p<0.05, **p<0.01, ***p<0.001 (Piscitelli et al., 2020)

**Table S5.** HR-MS. HR-MS instrumental parameters

| **Parameter** | **Hydroalcoholic fractions** | | **Lipophilic fractions** |
| --- | --- | --- | --- |
| Declustering potential (V) | 70 | 80 | |
| Collision energy (V) | 45 | 35 | |
| Collision energy spread (V) | 25 | 15 | |
| Ion spray voltage (V) | -4500 | -4500 | |
| Ion source heater (°C) | 600 | 600 | |
| Curtain gas (psi) | 35 | 35 | |
| Ion source gas (psi) | 45 | 45 | |


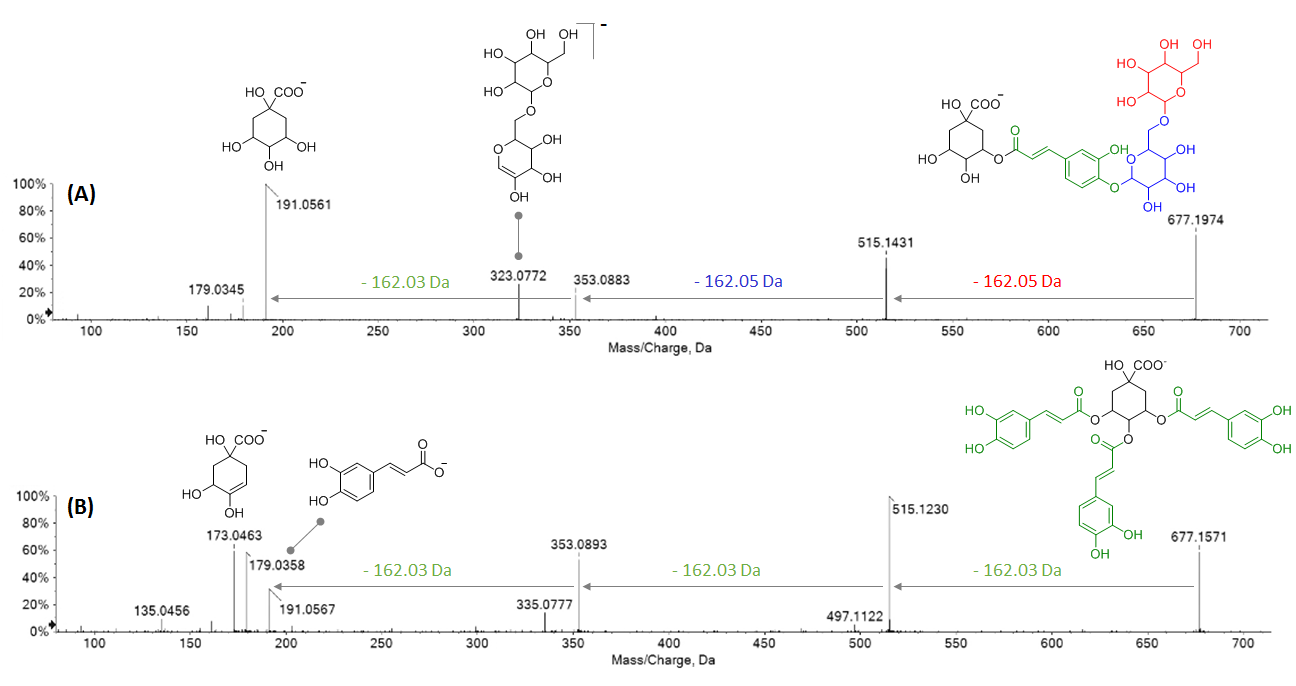


**Figure S1**. ToF/MS^2^ spectra of compounds (A) **13**, and (B) **49**. The software used to generate this figure was PeakView - Analyst TF 1.7.

**Figure S2.** Proposed fragmentation pathways of glycerol-phospholipids (**5’**, **7’** and **8’**) tentatively identified. Theoretical *m/z* values are reported below each structure. The software used to generate this figure was PeakView - Analyst TF 1.7.


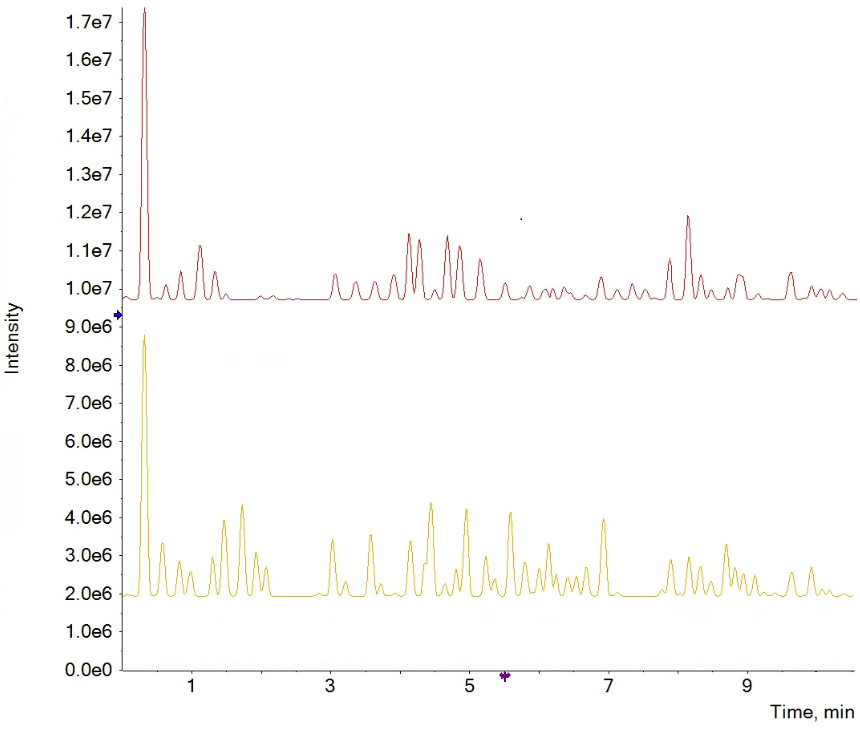


**Figure S3.** Representative TICs of hydro-alcoholic extracts from PR (in red color) and PG (in yellow color) samples. The software used to generate this figure was PeakView - Analyst TF 1.7.


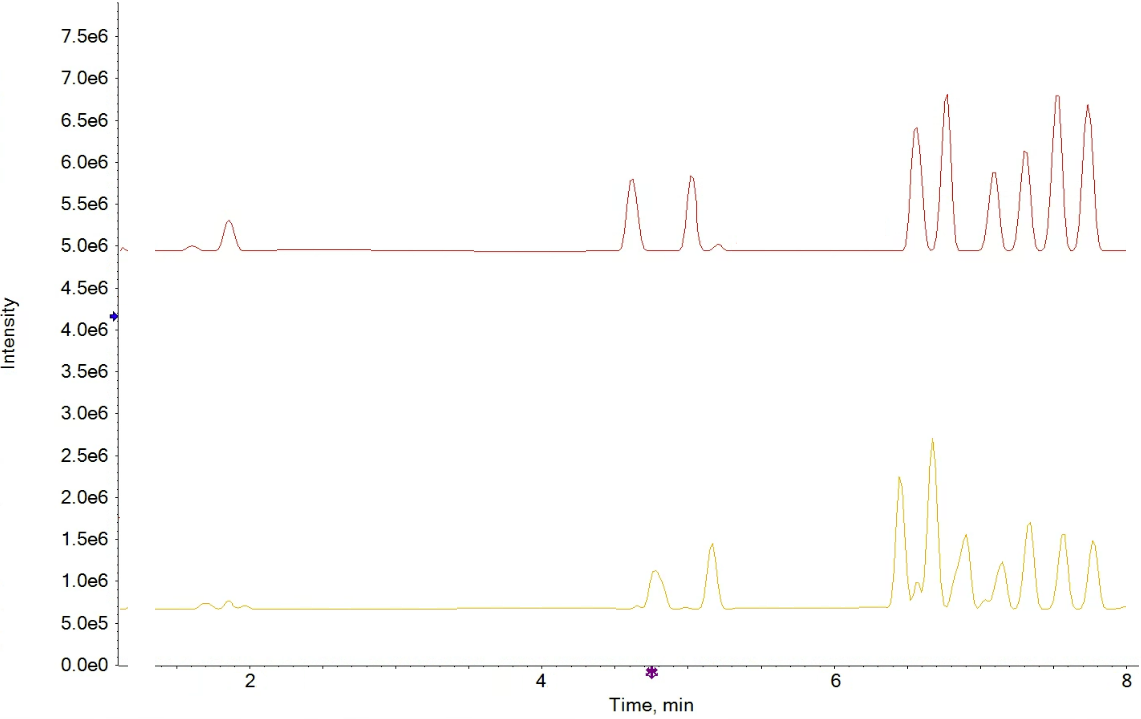


**Figure S4.** Representative TICs of lipophilic extracts from PR (in red color) and PG (in yellow color) samples. The software used to generate this figure was PeakView - Analyst TF 1.7.


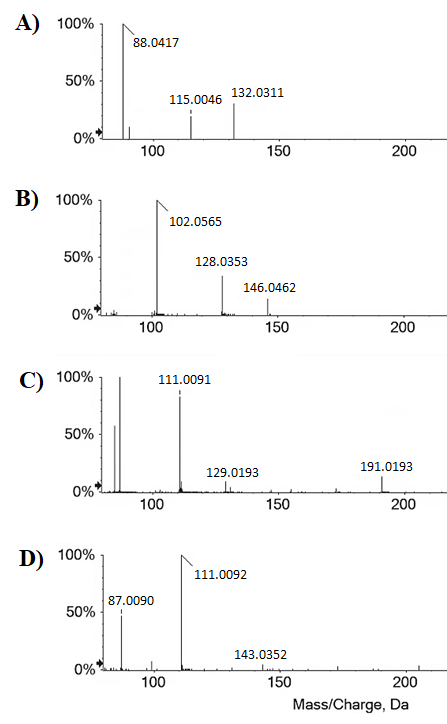


**Figure S5.** MS/MS spectra of non-phenolic compounds in hydro-alcoholic extracts: (A) 1; (B) 2; (C) 3; (D) 4. The software used to generate this figure was PeakView - Analyst TF 1.7.


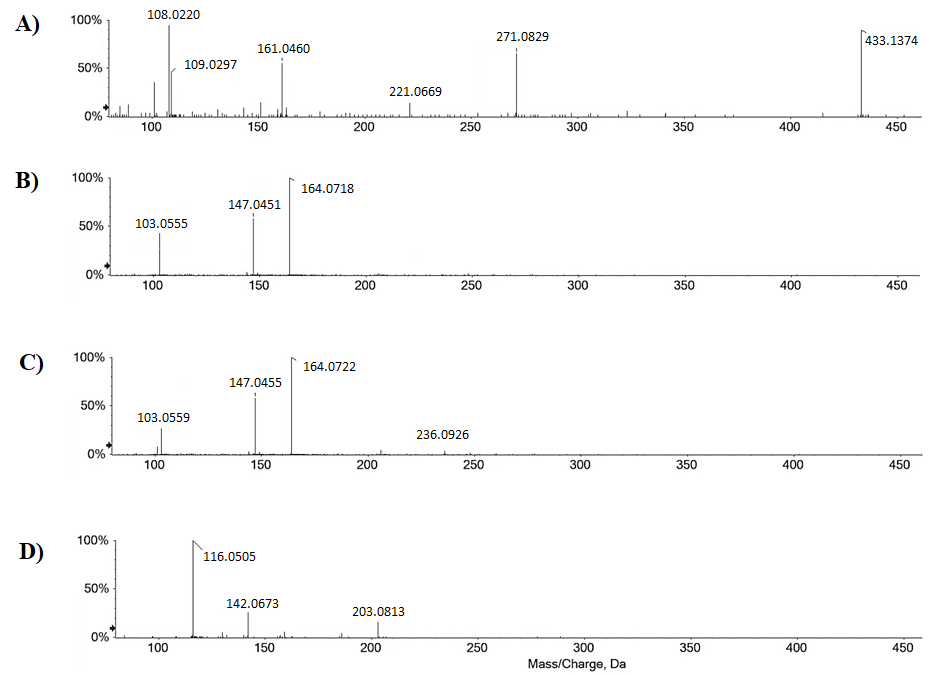


**Figure S6.** MS/MS spectra of non-phenolic compounds in hydro-alcoholic extracts: (A) 5; (B) 6; (C) 7; (D) 9. The software used to generate this figure was PeakView - Analyst TF 1.7.


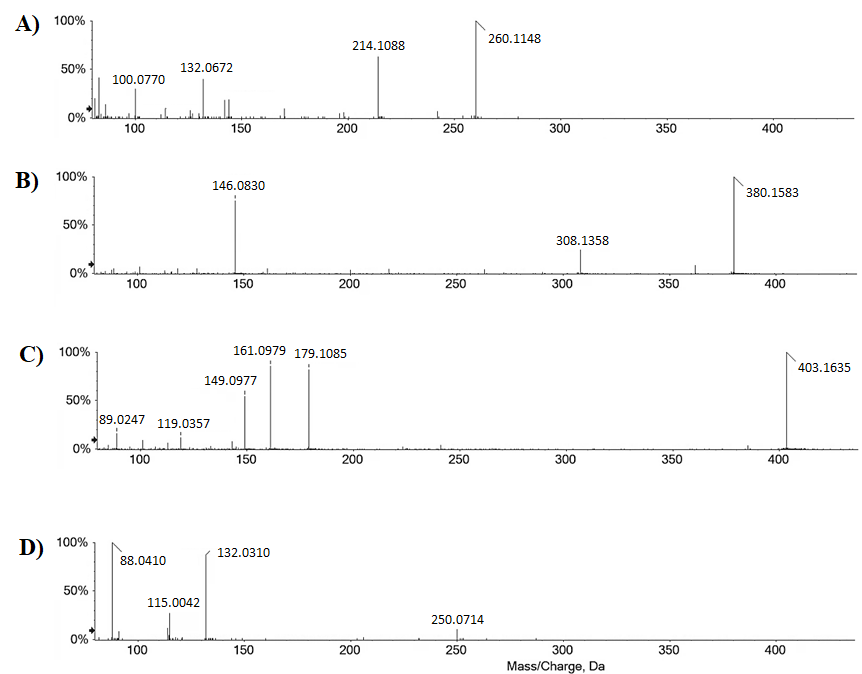


**Figure S7.** MS/MS spectra of non-phenolic compounds in hydro-alcoholic extracts: (A) 10; (B) 11; (C) 14; (D) 20. The software used to generate this figure was PeakView - Analyst TF 1.7.


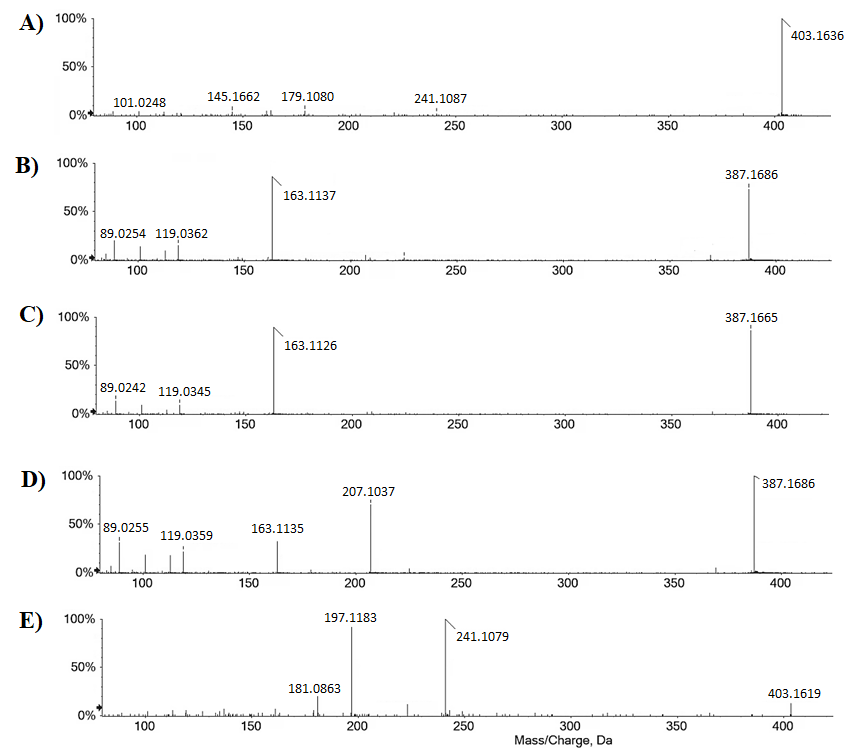


**Figure S8.** MS/MS spectra of non-phenolic compounds in hydro-alcoholic extracts: (A) 22; (B) 26; (C) 27; (D) 29; (E) 32. The software used to generate this figure was PeakView - Analyst TF 1.7.


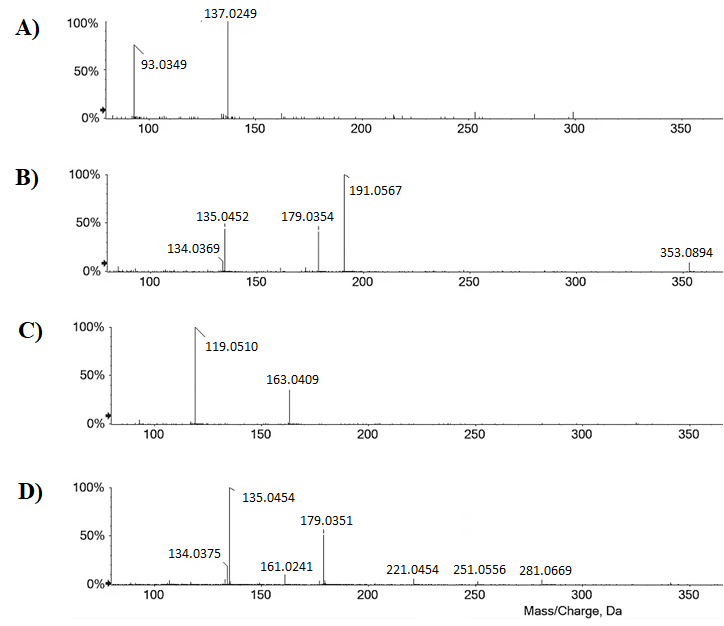


**Figure S9.** MS/MS spectra of phenolic compounds in hydro-alcoholic extracts: (A) 8; (B) 12; (C) 15; (D) 16. The software used to generate this figure was PeakView - Analyst TF 1.7.


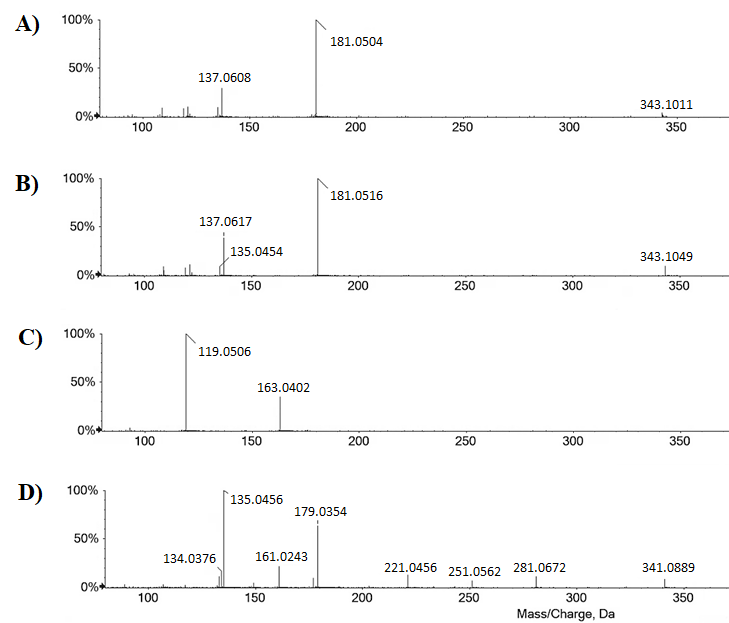


**Figure S10.** MS/MS spectra of phenolic compounds in hydro-alcoholic extracts: (A) 17; (B) 19; (C) 21; (D) 23. The software used to generate this figure was PeakView - Analyst TF 1.7.


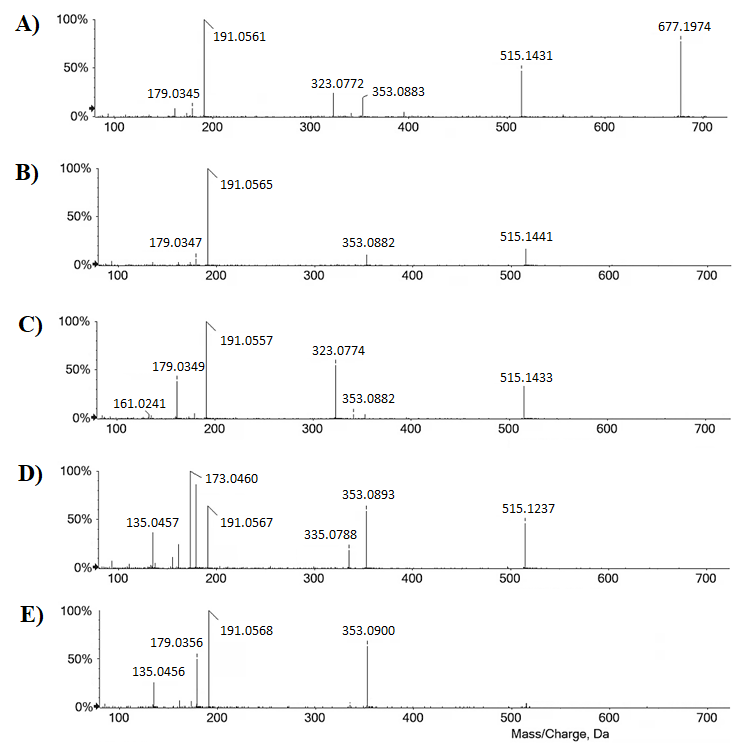


**Figure S11.** MS/MS spectra of phenolic compounds in hydro-alcoholic extracts: (A) 13; (B) 18; (C) 24; (D) 36; (E) 37. The software used to generate this figure was PeakView - Analyst TF 1.7.


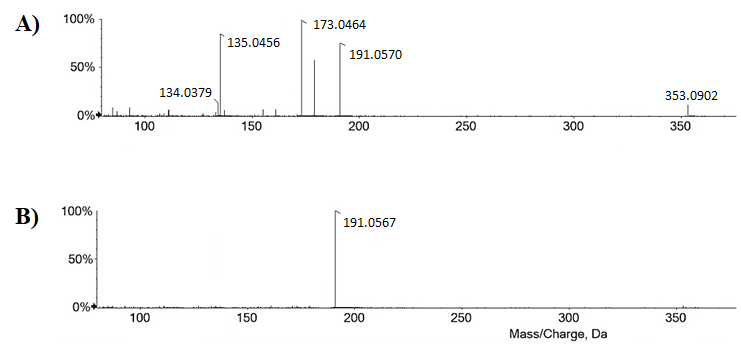


**Figure S12.** MS/MS spectra of phenolic compounds in hydro-alcoholic extracts: (A) 25; (B) 28. The software used to generate this figure was PeakView - Analyst TF 1.7.


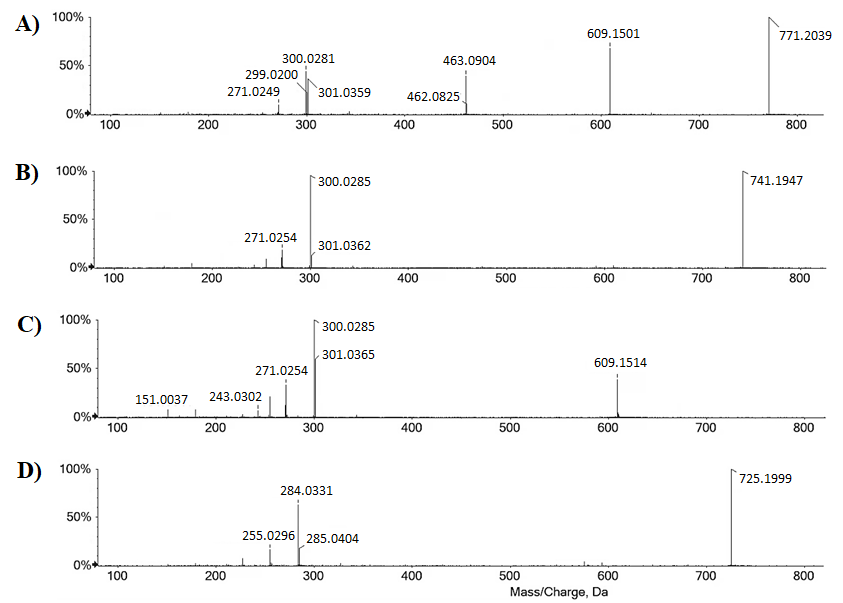


**Figure S13.** MS/MS spectra of phenolic compounds in hydro-alcoholic extracts: (A) 30; (B) 33; (C) 34; (D) 35. The software used to generate this figure was PeakView - Analyst TF 1.7.


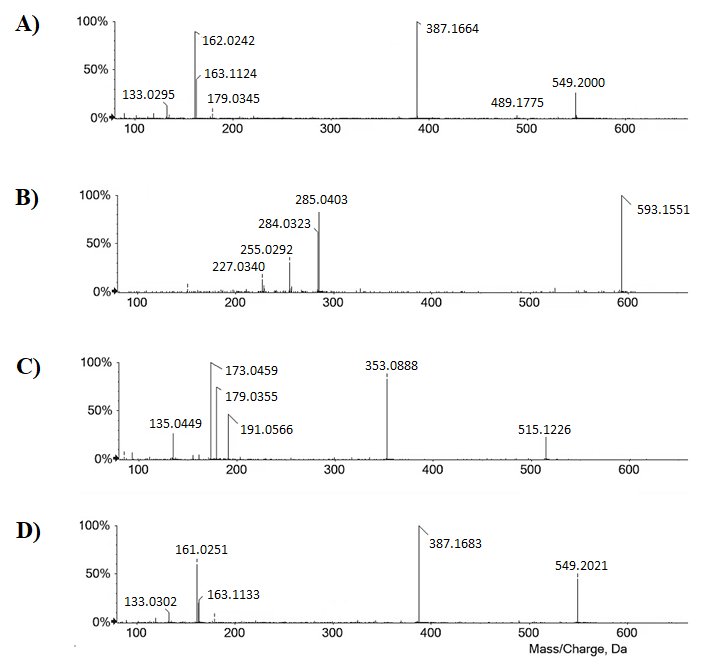


**Figure S14.** MS/MS spectra of phenolic compounds in hydro-alcoholic extracts: (A) 38; (B) 39; (C) 40; (D) 41. The software used to generate this figure was PeakView - Analyst TF 1.7.


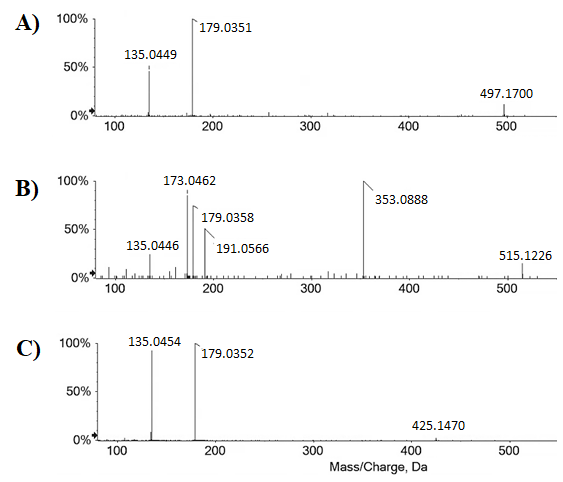


**Figure S15.** MS/MS spectra of phenolic compounds in hydro-alcoholic extracts: (A) 42; (B) 44; (C) 45. The software used to generate this figure was PeakView - Analyst TF 1.7.


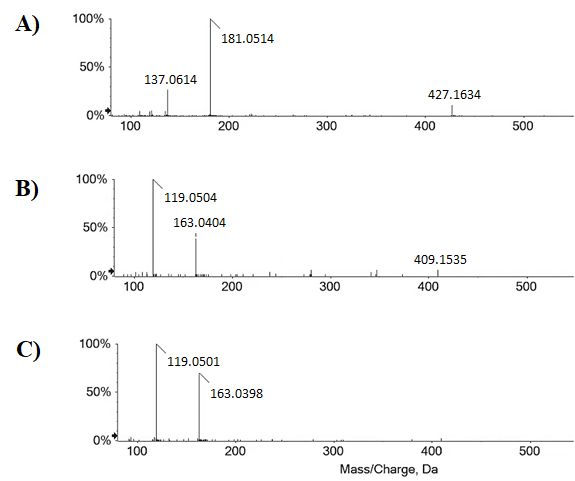


**Figure S16.** MS/MS spectra of phenolic compounds in hydro-alcoholic extracts: (A) 46; (B) 47; (C) 48. The software used to generate this figure was PeakView - Analyst TF 1.7.


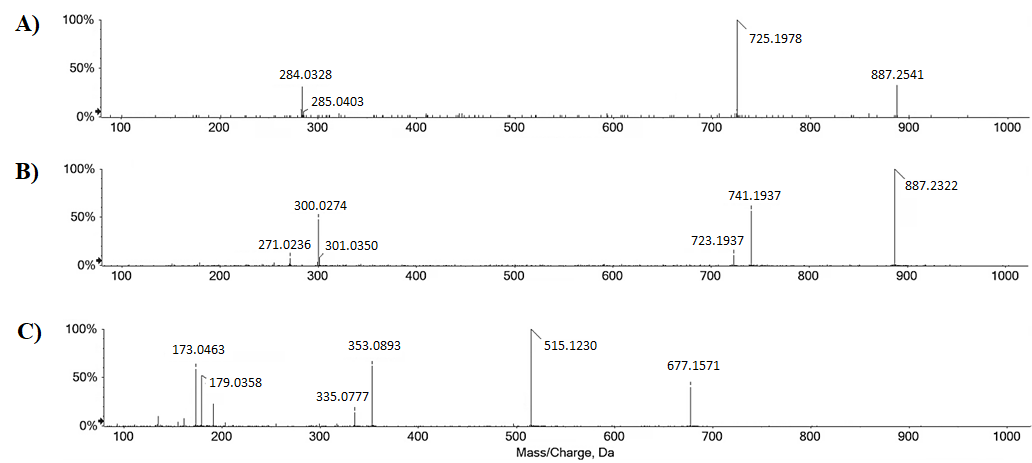


**Figure S17.** MS/MS spectra of phenolic compounds in hydro-alcoholic extracts: (A) 31; (B) 43; (C) 49. The software used to generate this figure was PeakView - Analyst TF 1.7.


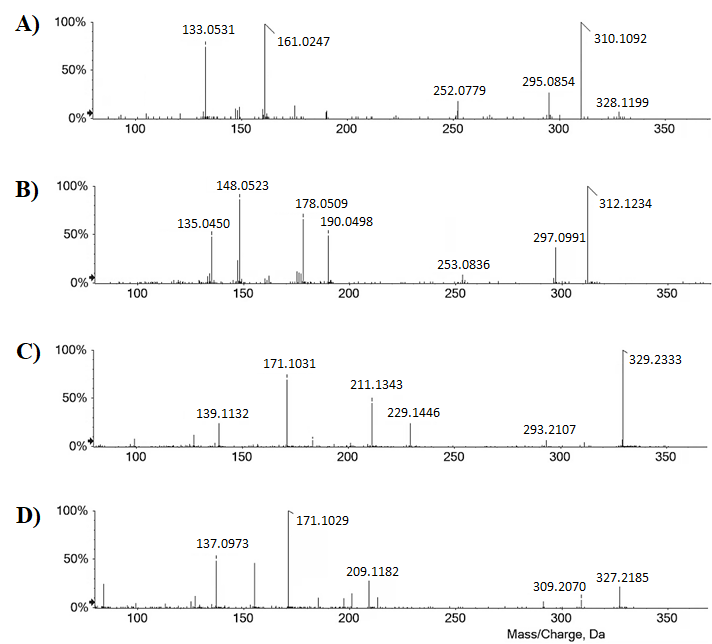


**Figure S18.** MS/MS spectra of compounds in lipophilic extracts: (A) 1’; (B) 2’; (C) 3’; (D) 4’. The software used to generate this figure was PeakView - Analyst TF 1.7.


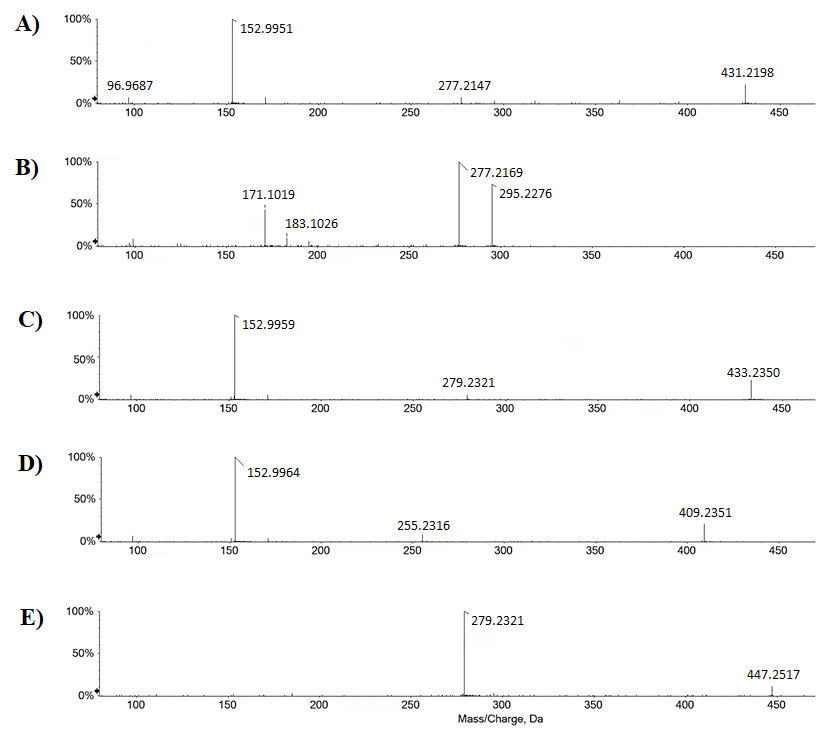


**Figure S19.** MS/MS spectra of compounds in lipophilic extracts: (A) 5’; (B) 6’; (C) 7’; (D) 8’; (E) 9’. The software used to generate this figure was PeakView - Analyst TF 1.7.


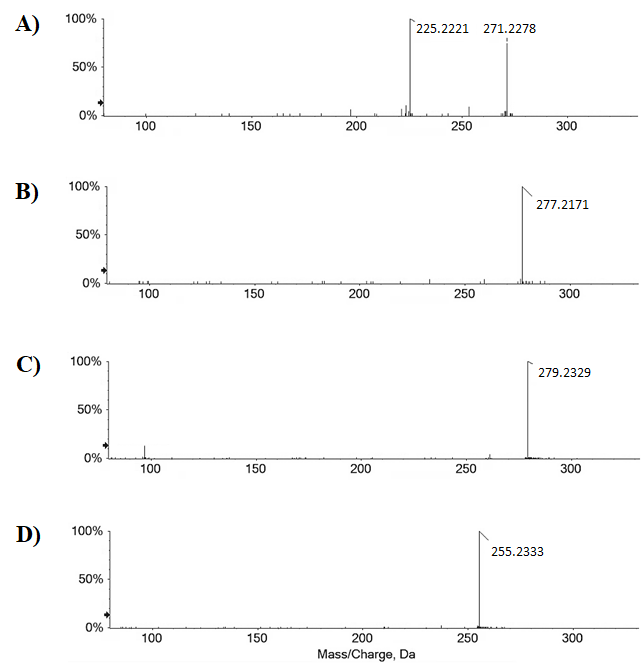


**Figure S20.** MS/MS spectra of compounds in lipophilic extracts: (A) 10’; (B) 11’; (C) 12’; (D) 13’. The software used to generate this figure was PeakView - Analyst TF 1.7.


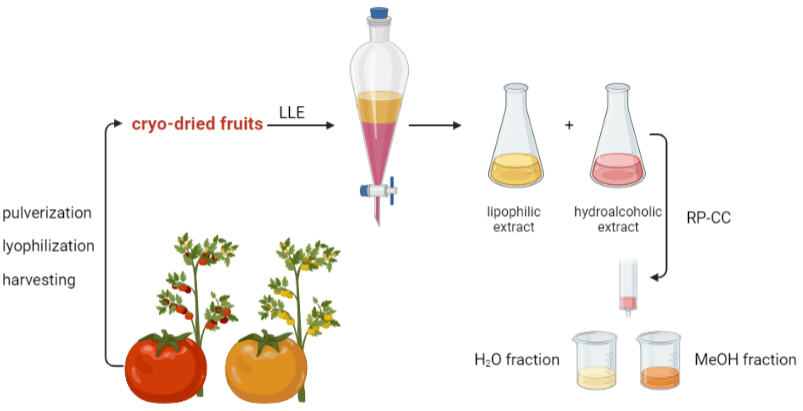


**Figure S21.** Extraction and fractionation scheme. Tomato fruits, after harvesting, were lyophilized and pulverized. Cryo-dried fruits underwent liquid-liquid extraction (LLE) obtaining a lipophilic chloroform extract, and an hydroalcoholic extract. This latter was fractionated to obtain an aqueous and a methanol fraction. The lipophilic extract was first dried over anhydrous Na_2_SO_4_ and then distilled under reduced pressure until chloroform was completely removed. The aqueous and alcoholic fractions were also dried using the rotary evaporator. Dried samples were then reconstituted in appropriate solvents for chemical and biological analyses.

**Scheme S1.** Chromatic Scheme. Chromatic Scheme of biological activities observed testing PG/PR-CTR and PG/PR- Cd, Cr, Pb on gastric and colorectal cancer cells. Statistical significance (p<0.05 – Dunnett’s test) from negative control was reported in grayscale.


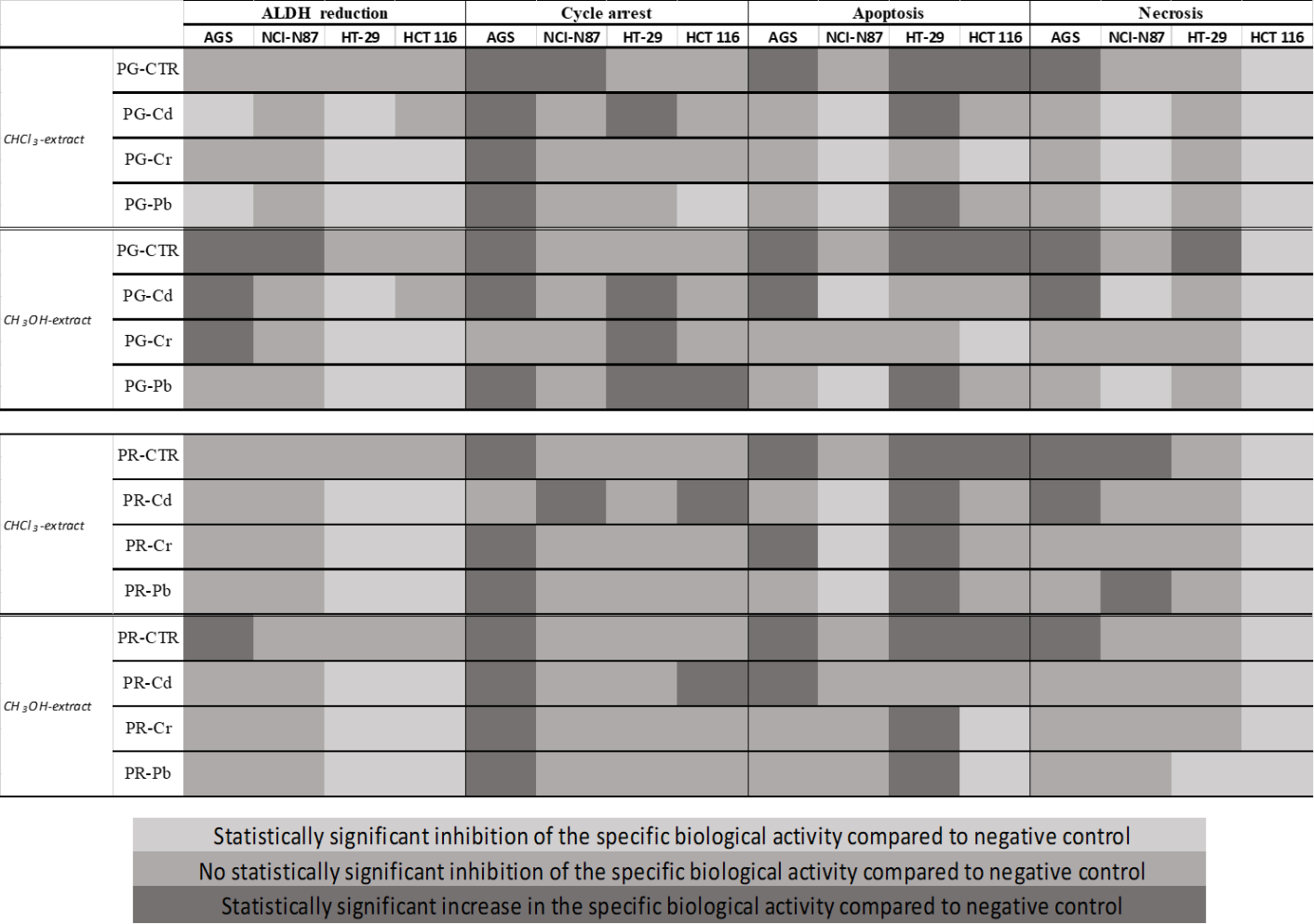


**References:**

Piscitelli C, Lavorgna M, De Prisco R, Coppola E, Grilli E, Russo C, Isidori M. Tomato plants (*Lycopersicon esculentum* L.) grown in experimental contaminated soil: bioconcentration of potentially toxic elements and free radical scavenging evaluation. Plos One. 2020; 15(8), 1-14, e0237031
